# Reduced striatal M4-cholinergic signaling following dopamine loss contributes to parkinsonian and l-DOPA–induced dyskinetic behaviors

**DOI:** 10.1126/sciadv.adp6301

**Published:** 2024-11-20

**Authors:** Beatriz E. Nielsen, Christopher P. Ford

**Affiliations:** ^1^ Department of Pharmacology, University of Colorado School of Medicine, Anschutz Medical Campus, Aurora, CO 80045, USA.; ^2^ Aligning Science Across Parkinson’s (ASAP) Collaborative Research Network, Chevy Chase, MD 20815, USA.

## Abstract

A dynamic equilibrium between dopamine and acetylcholine (ACh) is essential for striatal circuitry and motor function, as imbalances are associated with Parkinson’s disease (PD) and levodopa-induced dyskinesia (LID). Conventional theories posit that cholinergic signaling is pathologically elevated in PD as a result of increased ACh release, which contributes to motor deficits. However, using approaches to measure receptor-mediated signaling, we found that, rather than the predicted enhancement, the strength of cholinergic transmission at muscarinic M4 receptor synapses on direct pathway medium spiny neurons was decreased in dopamine-depleted mice. This adaptation was due to a reduced postsynaptic M4 receptor function, resulting from down-regulated receptors and downstream signaling. Restoring M4 transmission unexpectedly led to a partial alleviation of motor deficits and LID dyskinetic behavior, revealing an unexpected prokinetic effect in addition to the canonical antikinetic role of M4 receptors. These findings indicate that decreased M4 function differentially contributes to parkinsonian and LID pathophysiology, representing a promising target for therapeutic intervention.

## Teaser

M4-cholinergic signaling decreases following dopamine loss, affecting parkinsonian and levodopa-induced dyskinetic behavior.

## INTRODUCTION

Parkinson’s disease (PD) is a neurodegenerative movement disorder characterized by the progressive loss of dopamine (DA) neurons of the substantia nigra pars compacta (SNc), resulting in dopaminergic denervation of the dorsal striatum (DSt). The loss of DA drives motor impairment as a result of alterations in striatal circuits that ultimately lead to imbalances in output from the direct and indirect pathways, composed of medium spiny neurons expressing either D1 receptors (dMSNs) or D2 receptors (iMSNs), respectively (*1*, *2*). DA depletion is also believed to increase striatal acetylcholine (ACh) that arises mainly from local cholinergic interneurons (ChIs) (*3*–*6*). Hence, the DA-ACh balance hypothesis predicts that the loss of DA together with enhanced ACh are thought to be major pathological elements contributing to parkinsonian motor dysfunction (*3*–*10*). However, despite the importance of ACh in the striatum, how DA depletion specifically alters cholinergic transmission and receptor activation remains unclear, as conflicting results for changes in ChI excitability and ACh levels following the loss of DA have been reported in different models of PD (*5*, *8*, *11*–*19*).

In addition to the circuit effects of striatal nicotinic receptors, direct modulation of MSNs by ACh occurs through postsynaptic G protein–coupled muscarinic receptors. While G_q_-coupled M1 receptors are expressed in all MSNs, G_i/o_-coupled M4 receptors are predominantly expressed in dMSNs (*20*, *21*). M4 receptors have been regarded as the primary muscarinic subtype involved in DA-ACh interactions by decoding ChI firing patterns (*22*) and regulating DA transmission and associated motor behaviors (*23*). M4 receptors are believed to participate in the control of motor function by modulating the presynaptic release of DA (*24*, *25*) and by postsynaptically opposing G_olf/s_-coupled D1 receptor signaling, which regulates the induction of corticostriatal synaptic plasticity (*26*–*29*). Thus, according to the DA-ACh balance hypothesis in the context of PD, the predicted increase in ACh levels following DA loss is thought to translate into enhanced M4 signaling and reduced D1 signaling in dMSNs, resulting in an overall inhibition of the direct pathway and the suppression of movement (*3*, *4*, *6*). To counteract the depletion of DA, the standard PD treatment uses the precursor levodopa [3,4-dihydroxy-l-phenylalanine (l-DOPA)], which eventually leads to the onset of abnormal involuntary movements (AIMs) referred to as levodopa-induced dyskinesia (LID) as the disease progresses (*30*). However, to date, directly determining how M4 signaling is altered following DA loss in PD or following l-DOPA therapy in LID has yet to be addressed.

Here, we examined direct pathway M4-mediated cholinergic signaling in DA-depleted mice and found that rather than being increased, the strength of transmission was decreased as a result of diminished postsynaptic M4 receptor function in dMSNs. This cell-subtype and synapse-specific adaptation in response to dopaminergic degeneration had broad implications at circuit level depending dynamically on DA levels because restoring aberrant M4 signaling unexpectedly alleviated aspects of both parkinsonian motor deficits and levodopa-induced dyskinetic behaviors. This reveals that a reduced direct pathway M4 transmission may play a potential and previously unnoticed role in the pathophysiology of PD and the progression to LID.

## RESULTS

### Striatal M4-mediated cholinergic transmission is reduced following DA depletion

**Fig. 1.**
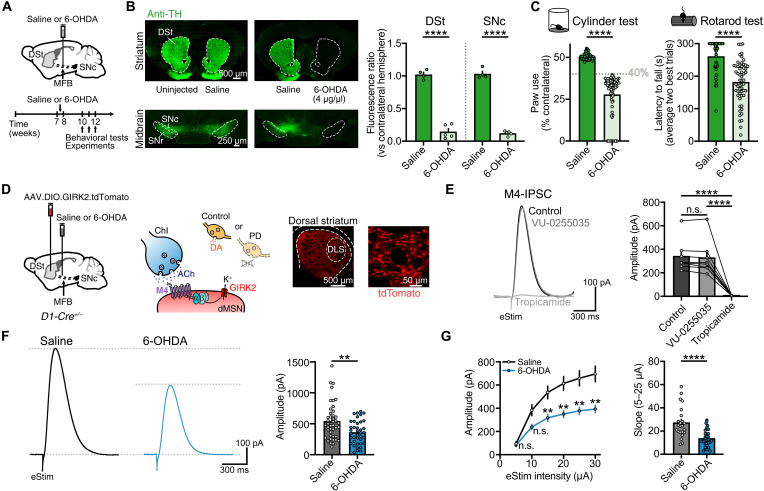
DA depletion reduces M4-mediated cholinergic synaptic transmission. (**A**) Schematics of saline or 6-OHDA injections into the MFB and experiments timeline. (**B**) TH immunoreactivity of striatal and midbrain sections following saline or 6-OHDA injections (left) and quantification in DSt and SNc (right) (saline: *N* = 4, 6-OHDA: *N* = 5; *P* < 0.0001; unpaired *t* test). (**C**) Summary data of cylinder test performance (left) (saline: *N* = 60, 6-OHDA: *N* = 63; *P* < 0.0001; Mann-Whitney) for all mice included in Figs. 1 and 2 of this study. The cutoff value used as inclusion criteria for experimental groups is shown on the bar chart. Summary data of rotarod test performance (right) (saline: *N* = 42, 6-OHDA: *N* = 57; *P* < 0.0001; Mann-Whitney). (**D**) Schematics of AAV9.hSyn.DIO.tdTomato.T2A.GIRK2 and saline/6-OHDA injections into DSt and MFB, respectively, in D1-Cre mice (left). ChI-dMSNs synapse showing the intracellular coupling between M4 receptor and GIRK2 (center). tdTomato fluorescence in striatal section and close-up view of tdTomato^+^ dMSNs (right). (**E**) Representative traces and quantification of M4-IPSCs following application of M1 receptor (VU-0255035) and M4 receptor (tropicamide) selective antagonists (*n* = 8, *N* = 3; *P* < 0.0001; repeated measures (RM) one-way ANOVA and Holm-Šídák’s post hoc test). (**F**) Representative traces and quantification of electrically evoked M4-IPSCs (saline: *n* = 43, *N* = 16; 6-OHDA: *n* = 39, *N* = 13; *P* = 0.0045; Mann-Whitney). (**G**) Plot of M4-IPSC amplitudes versus electrical stimulation intensity (saline: *n* = 23, *N* = 12; 6-OHDA: *n* = 35, *N* = 12; *P* = 0.0002 for treatment effect; mixed-model ANOVA; Šídák’s post hoc test) and summary data of slope for 5 to 25 μA in range (*P* < 0.0001; Mann-Whitney). Summary data are means ± SEM. Extended statistical data are provided in table S1. *n*: number of cells, *N*: number of mice; n.s. (not significant), *P* > 0.05; *P < 0.05; **P < 0.01; and ****P < 0.0001.

Mice were injected with a high dose (4 μg) of 6-hydroxydopamine (6-OHDA) unilaterally into the medial forebrain bundle (MFB) to induce a near complete degeneration of striatal DA inputs, while control animals were similarly injected with saline (Fig. 1, A and B). Three to 4 weeks after 6-OHDA injection, tyrosine hydroxylase (TH) immunoreactivity was reduced in the DSt and SNc (Fig. 1B). As expected, lesioning the DA system resulted in motor impairment when assayed with cylinder and rotarod tests (Fig. 1C). The presence of motor deficits in the cylinder test was used as the criteria for including mice in parkinsonian experimental groups for the rest of the study, with a contralateral paw use below 40% set as a cutoff (Fig. 1C).

Although the loss of DA has been predicted to drive increases in striatal ACh levels (*3*, *4*, *6*), how this translates into altered cholinergic transmission remains unclear. Thus, we set out to examine changes in ACh transmission onto dMSNs at M4 receptor synapses in the dorsolateral striatum (DLS) of DA-lesioned animals. As M4 receptors do not efficiently couple to endogenous ion channels in dMSNs, directly measuring the strength of direct pathway M4 receptor signaling in real time and in intact tissue has been hindered due to a lack of easily detected synaptic responses. To overcome this, we selectively expressed exogenous G protein–coupled inwardly rectifying K^+^ channels (GIRK2; Kir3.2) in dMSNs, where they can couple to endogenous M4 receptors so that the resulting potassium current provides an electrophysiological readout of receptor activation (Fig. 1D) (*22*, *31*). To overexpress GIRK2 channels in dMSNs, a Cre-dependent virus encoding for GIRK2 and tdTomato fluorophore (AAV.DIO.GIRK2.T2A.tdTomato) was unilaterally injected into the DSt of D1-Cre (*Drd1-Cre^+/−^*) mice at the same time as either 6-OHDA or saline was injected into the MFB (Fig. 1D). Three weeks following, brain slices were prepared and recordings made from dMSNs in the DLS. The overexpression of GIRK2 in dMSNs did not alter basic membrane properties (fig. S1A) or motor behavioral performance (fig. S1B). In the presence of antagonists to block glutamate, γ-aminobutyric acid (GABA), DA, and nicotinic receptors to isolate muscarinic transmission, a single electrical stimulation (25 μA, 0.5 ms) evoked M4 receptor–mediated inhibitory postsynaptic currents (M4-IPSCs) in tdTomato^+^-GIRK2^+^ dMSNs (Fig. 1E and fig. S1C). These currents were absent in GIRK2^+^ iMSNs (figs. S1, D and E) (*20*, *21*) and unaffected by the M1 antagonist, VU-0255035 (1 μM) but blocked by the M4 antagonist, tropicamide (1 μM) (Fig. 1E). Unexpectedly, the amplitude of M4-IPSCs was significantly reduced in 6-OHDA–treated mice (Fig. 1F). Similar results could be seen over a range of electrical stimuli intensities (5 to 30 μA, 0.5 ms) and examining input/output relationships across conditions (Fig. 1G). These results indicate that cholinergic transmission onto dMSNs through M4 receptors is reduced following the degeneration of dopaminergic inputs.

As ACh levels, and therefore cholinergic signaling, are also regulated by AChE-mediated hydrolysis, we tested whether the enzymatic clearance was increased following DA loss by applying the AChE inhibitor ambenonium (10 nM) while recording electrically evoked M4-IPSCs in GIRK2-expressing dMSNs (fig. S1G). Because AChE limits the duration of M4-mediated transmission (*22*), the net charge transfer of evoked synaptic currents were increased following AChE inhibition (fig. S1G). However, there was no difference in the effect of ambenonium after DA depletion (fig. S1G), suggesting that there were no functional changes in cholinesterase-mediated ACh clearance.

### Reduced M4-mediated cholinergic transmission in response to DA loss is due to a decreased postsynaptic M4 function

**Fig. 2.**
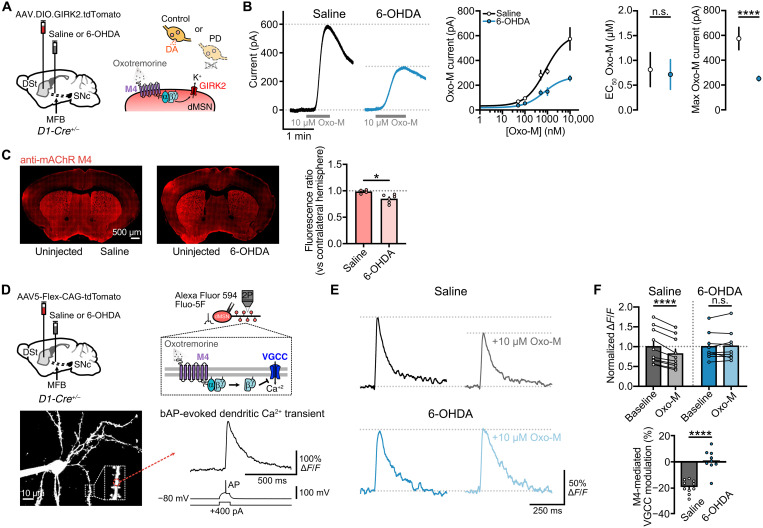
DA depletion reduces postsynaptic M4 receptor function in dMSNs. (**A**) Schematics of AAV9.hSyn.DIO.tdTomato.T2A.GIRK2 and saline/6-OHDA injections into DSt and MFB respectively in D1-Cre mice (left). Cartoon showing the bath application of Oxo-M (right). (**B**) Representative traces of M4-mediated Oxo-M currents following bath application of Oxo-M (10 μM). Spontaneous M4-IPSCs and electrical artifacts were blanked for clarity (left). Oxo-M concentration-response curves for M4 receptor (center). EC_50_ (median effective concentration) values from concentration-response curves (saline: *n* = 54, *N* = 5 to 12; 6-OHDA: *n* = 42, *N* = 5 to 9; *P* = 0.8348; unpaired *t* test) and maximal Oxo-M current values (right) (saline: *n* = 12, *N* = 10; 6-OHDA: *n* = 11, *N* = 9; *P* < 0.0001; Mann-Whitney). (**C**) M4 immunoreactivity in striatal sections following unilateral injections of saline and 6-OHDA into the MFB (left). Quantification is shown on the right (saline: *N* = 4, 6-OHDA: *N* = 6; *P* = 0.0139; unpaired *t* test). (**D**) Schematics of AAV5-Flex-CAG-tdTomato and saline/6-OHDA injections into DSt and MFB, respectively, in D1-Cre mice (top left) and cartoon depicting M4-mediated modulation of VGCC (top right). Two-photon image of TdTomato^+^ dMSN filled with Alexa Fluor 594 and representative trace of a bAP-evoked Ca^2+^ transient measured at its dendrite (bottom). (**E**) Representative dendritic Ca^2+^ transients for saline and 6-OHDA conditions before and after Oxo-M application. (**F**) Quantification of dendritic Ca^2+^ transients peak before and after Oxo-M application (top) (saline: *n* = 10, *N* = 5; *P* < 0.0001; paired *t* test/6-OHDA: *n* = 9, *N* = 4; *P* = 0.6206; paired *t* test) and percent M4-mediated modulation of VGCC for both conditions (bottom) (*P* < 0.0001; unpaired *t* test). Summary data are means ± SEM. Extended statistical data are provided in table S1. n.s., *P* > 0.05; *P < 0.05; and ****P < 0.0001.

To examine whether the decrease in M4-IPSCs was due to a postsynaptic change in the strength of M4 receptor function, we bath applied the muscarinic agonist oxotremorine-M (Oxo-M) to generate concentration-response curves from M4-mediated outward currents in GIRK2-expressing dMSNs (Fig. 2, A and B). While the sensitivity of M4 receptor remained unchanged in 6-OHDA–treated mice, postsynaptic efficacy was reduced (Fig. 2B), indicating that the reduction in M4-IPSCs following DA loss likely resulted from decreased M4 receptor signaling. The decrease in M4-mediated currents was not due to differences in GIRK2 expression, as GIRK2 protein levels were similar in all conditions (fig. S2A). Instead, the reduction was at least partially due to a decrease in M4 receptor expression, as we found a ~20% reduction in total striatal M4 immunoreactivity in mice unilaterally injected with 6-OHDA compared to saline-treated controls (Fig. 2C).

To determine whether the strength of M4 receptor mediated transmission in dMSNs also occurs in a more progressive animal model of PD, we examined potential changes in MitoPark mice (*32*). This mouse model consists of a selective knockout of the gene encoding for mitochondrial transcription factor A (Tfam) from DA neurons, which leads to mitochondrial dysfunction and gradual degeneration (*32*). We crossed Tfam^fl/fl^ and DAT-Cre mice generating MitoPark mice (*Tfam^fl/fl^; DAT-Cre^+/−^*) and littermate controls (*Tfam^fl/fl^; DAT-Cre^−/−^*) (fig. S2B). A non-Cre–dependent virus encoding for GIRK2 (AAV.GIRK2.T2A.tdTomato) was injected in the DSt at 6 to 7 months (fig. S2B) because mice at this age show substantial DA depletion similar to 6-OHDA–lesioned mice (*32*). After 3 weeks of GIRK2 expression, recordings were again made from DLS dMSNs, which were identified by the presence of M4-IPSCs. The amplitude of electrically evoked M4-IPSCs and the maximum outward currents produced by Oxo-M were reduced in MitoPark mice compared to their littermate controls (fig. S2, C and D). Thus, the strength of direct pathway M4 signaling is reduced following DA loss in both acute and progressive depletion models.

In addition to examining M4 function through the activation of exogenously expressed GIRK2 channels, we also determined receptor signaling to endogenous effectors by measuring the M4 receptor–mediated inhibition of dendritic Ca^2+^ entry through voltage-gated calcium channels (VGCCs) activated by backpropagating action potentials (bAPs) (Fig. 2D) (*33*, *34*). dMSNs in the DLS were identified in D1-Cre mice by selective viral expression of TdTomato, and current-clamp recordings were made using electrodes filled with Alexa Fluor 594 (10 μM) to visualize dendritic arbor morphology and Fluo-5F (100 μM) to enable the detection of bAP-evoked dendritic Ca^2+^ transients (Fig. 2D). Single bAPs were elicited by somatic depolarizing current injection (200 to 400 pA, 100 ms), and Ca^2+^ transients were measured at dendritic shafts or spines located at <60 μm from soma by two-photon point scanning (Fig. 2D). In the presence of the selective M1 antagonist VU-0255035 (500 nM) (in addition to blockers for glutamate, DA, GABA and nicotinic receptors), application of Oxo-M (10 μM) reduced the amplitude of Ca^2+^ transients by ~20% in control saline-treated condition but had no effect in dMSNs from 6-OHDA–treated animals (Fig. 2, E and F), confirming that dendritic direct pathway postsynaptic M4 receptor signaling is reduced in response to DA depletion. The near complete reduction in M4 function, as opposed to the ~40% reduction when using GIRK2 as a readout, may reflect signaling differences between the effectors used to record M4 receptor activity or across dMSNs subcellular compartments.

### Overexpression of M4 receptors in dMSNs restores cholinergic transmission and ameliorates balance and coordination parkinsonian motor deficits

**Fig. 3.**
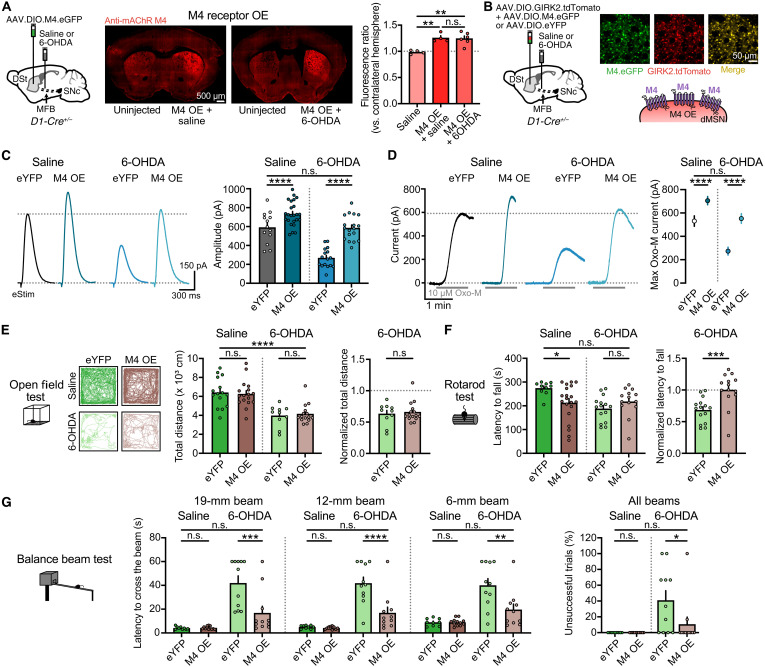
Overexpression of M4 receptors in dMSNs restores cholinergic transmission and ameliorates motor deficits in parkinsonian mice. (**A**) Schematics and M4 immunoreactivity for M4 overexpression (left, center). Fluorescence quantification (right) (saline data from Fig. 2C) (saline: *N* = 4; M4 saline: *N* = 4; M4 6-OHDA: *N* = 6; *P* = 0.0017; one-way ANOVA and Šídák). (**B**) Schematics and fluorescence images of M4 or eYFP and GIRK2 expression. (**C**) Representative traces and quantification of electrically evoked M4-IPSCs (eYFP saline: *n* = 13, *N* = 6; M4 saline: *n* = 24, *N* = 8; eYFP 6-OHDA: *n* = 16, *N* = 5; M4 6-OHDA: *n* = 18, *N* = 7; *P* < 0.0001 for treatment and group effects; two-way ANOVA and Šídák). (**D**) Representative traces and quantification of maximum Oxo-M currents (eYFP saline: *n* = 13, *N* = 6; M4 saline: *n* = 20, *N* = 10; eYFP 6-OHDA: *n* = 10, *N* = 5; M4 6-OHDA: *n* = 9, *N* = 5; *P* < 0.0001 for treatment and group effects; two-way ANOVA and Šídák. (**E**) Example traces and quantification of open field movement (eYFP saline: *N* = 15; M4 saline: *N* = 17; eYFP 6-OHDA: *N* = 10; M4 6-OHDA: *N* = 15; *P* < 0.0001 for treatment and *P* = 0.9574 for group effects; two-way ANOVA and Šídák. Normalized data: *P* = 0.6433; Mann-Whitney). (**F**) Latency to fall from rotarod (eYFP saline: *N* = 10; M4 saline: *N* = 20; eYFP 6-OHDA: *N* = 15; M4 6-OHDA: *N* = 14; *P* = 0.0158 for treatment and *P* = 0.341 for group effects; two-way ANOVA and Šídák. Normalized data: *P* = 0.0006; Mann-Whitney). (**G**) Latency to cross (left) and percentage of unsuccessful trials (right) in balance beam test (eYFP saline: *N* = 9; M4 saline: *N* = 12; eYFP 6-OHDA: *N* = 11; M4 6-OHDA: *N* = 11; two-way ANOVA and Šídák) (19-mm beam: *P* < 0.0001 for treatment and *P* = 0.0055 for group effects/12-mm beam: *P* < 0.0001 for treatment and *P* = 0.0009 for group effects/6-mm beam: *P* < 0.0001 for treatment and *P* = 0.0113 for group effects/unsuccessful trials: *P* = 0.0021 for treatment and *P* = 0.0599 for group effects). Summary data are means ± SEM. Extended statistical data are provided in table S1. n.s., *P* > 0.05; *P < 0.05; **P < 0.01; ***P < 0.001; and ****P < 0.0001.

In an attempt to revert the impairment in transmission, we designed an Adeno-associated virus (AAV) encoding for the Cre-dependent expression of the M4 receptor and a fluorescent reporter enhanced green fluorescent protein (eGFP) to test whether selective overexpression in dMSNs could rescue deficits in cholinergic signaling in parkinsonian mice. Three weeks following injection of AAV.DIO.M4.eGFP into the DSt of D1-Cre mice, M4 immunoreactivity was increased by ~25% in the injected hemisphere of both saline and 6-OHDA–treated mice (Fig. 3A). Co-injection of AAV.DIO.M4.eGFP together with AAV.DIO.GIRK2.T2A.tdTomato in D1-Cre mice led to widespread eGFP and tdTomato fluorescence in overlapping populations of neurons (Fig. 3B). Recordings from eGFP^+^/tdTomato^+^ dMSNs revealed that the amplitude of both M4-IPSCs (Fig. 3C) and M4-mediated outward currents evoked by Oxo-M (10 μM) (Fig. 3D) was greater in M4-overexpressing mice than in control mice [enhanced yellow fluorescent protein (eYFP)], in which AAV.DIO.GIRK2.T2A.tdTomato was co-injected with a control fluorophore (AAV.DIO.eYFP) (fig. S3A). Thus, overexpressed M4 receptors were functional and coupled to downstream effectors to mediate postsynaptic responses in dMSNs. Overexpression of M4 receptors in dMSNs had no effect on ChI excitability (fig. S3B).

We next tested whether selective M4 overexpression in dMSNs could limit the decrease in cholinergic transmission following DA loss. In agreement with our previous results, the amplitude of M4-IPSCs in control eYFP mice was reduced following 6-OHDA treatment (Fig. 3C). This deficit, however, was rescued in 6-OHDA–treated mice overexpressing M4 receptors such that despite complete DA depletion, the amplitude of M4-IPSCs was similar to saline-treated eYFP controls (Fig. 3C). The rescue of M4 transmission in parkinsonian mice could be seen across a range of electrical stimulation intensities (fig. S3C) and also when examining M4-mediated outward currents evoked by bath application of Oxo-M (Fig. 3D). Thus, the selective overexpression of M4 receptors in dMSNs was sufficient to restore the impairment in M4-mediated cholinergic transmission that followed DA depletion back to normal levels.

Although M4 signaling is thought to play an antikinetic role due to inhibition of the direct pathway (*23*, *26*), facilitatory mechanisms have also been described (*35*, *36*). To examine this, we looked at the impact of restoring cholinergic transmission on motor behavior by overexpressing M4 receptors in dMSNs in 6-OHDA–treated mice. Unexpectedly, we found that M4 overexpression had no effect on spontaneous locomotor activity and forelimb use in control or 6-OHDA–treated mice (Fig. 3E and fig. S3, D and E), but it improved the motor performance of parkinsonian mice in tasks testing balance and coordination, such as rotarod and balance beam (Fig. 3, F and G, and fig. S3F). In the rotarod task, the motor improvement was revealed after normalizing the latency to fall because overexpression of M4 receptors in control condition led to a basal impairment in the performance (Fig. 3F), likely due to an overinhibition of the direct pathway (*23*, *26*). In contrast, M4 overexpression did not have any effect on basal performance for the balance beam test so that the rescue in the 6-OHDA–induced deficits could be directly seen as a reduction in the latency to cross the beam and a decrease in foot slips despite the complete DA lesion (Fig. 3G and fig. S3F). In addition, as DA depletion reduces engagement in walking tasks, parkinsonian mice often remain immobile or stop while attempting to cross the beam, thus failing to complete a successful balance beam trial. This “start hesitation” behavior in mice has been compared to the “freezing of gait” observed in patients with PD, although it may reflect motivational deficits instead (*37*, *38*). As opposed to 6-OHDA–treated mice, which frequently stop, M4-overexpressing mice showed reduced hesitation to initiate movement, so that they succeeded in crossing the beam as frequently as eYFP-expressing saline control mice (Fig. 3G). Together, these results highlight that 6-OHDA–induced reduction in M4 function may contribute to aspects of parkinsonian motor impairment because restoring M4-mediated cholinergic transmission by selectively overexpressing M4 receptors in dMSNs improved balance and coordination performance without further impairing locomotion.

### Selective ablation of RGS4 from dMSNs rescues M4 transmission and alleviates parkinsonian motor deficits

**Fig. 4.**
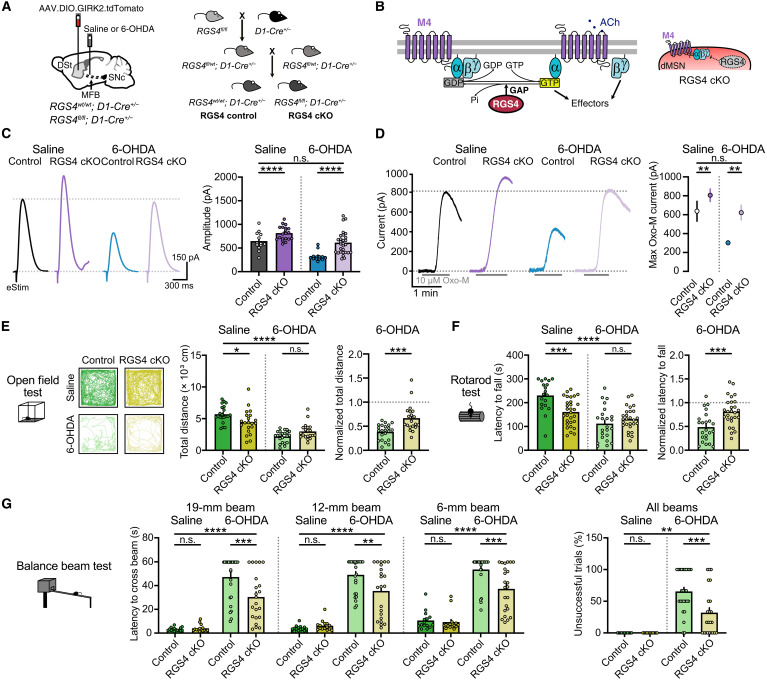
Selective ablation of RGS4 in dMSNs rescues M4 transmission and alleviates motor impairment in parkinsonian mice. (**A**) Schematics for GIRK2 expression in RGS4 control and RGS4 cKO mice. (**B**) Schematic of regulation of M4 signaling by RGS4 and illustration of RGS4 ablation in in dMSNs. (**C**) Representative traces and quantification of electrically evoked M4-IPSCs (control saline: *n* = 13, *N* = 7; RGS4 cKO saline: *n* = 24, *N* = 11; control 6-OHDA: *n* = 13, *N* = 6; RGS4 cKO 6-OHDA: *n* = 29, *N* = 10; *P* < 0.0001 for treatment and group effects; two-way ANOVA and Šídák). (**D**) Representative traces and quantification of maximum Oxo-M currents (control saline: *n* = 6, *N* = 5; RGS4 cKO saline: *n* = 15, *N* = 10; control 6-OHDA: *n* = 8, *N* = 5; RGS4 cKO 6-OHDA: *n* = 9, *N* = 7; *P* = 0.0028 for treatment and *P* = 0.0032 for group effects; two-way ANOVA and Šídák). (**E**) Example traces and quantification of open field movement (control saline: *N* = 21; RGS4 cKO saline: *N* = 21; control 6-OHDA: *N* = 22; RGS4 cKO 6-OHDA: *N* = 20; *P* < 0.0001 for treatment and *P* = 0.4591 for group effects; two-way ANOVA and Šídák. Normalized data: *P* = 0.0002; Mann-Whitney). (**F**) Latency to fall from rotarod (control saline: *N* = 19; RGS4 cKO saline: *N* = 30; control 6-OHDA: *N* = 23; RGS4 cKO 6-OHDA: *N* = 27; *P* < 0.0001 for treatment and *P* = 0.0407 for group effects; two-way ANOVA and Šídák. Normalized data: *P* = 0.0004; unpaired *t* test). (**G**) Latency to cross (left) and percentage of unsuccessful trials (right) in balance beam test (control saline: *N* = 15; RGS4 cKO saline: *N* = 15; control 6-OHDA: *N* = 26; RGS4 cKO 6-OHDA: *N* = 22–23; two-way ANOVA and Šídák) (19-mm beam: *P* < 0.0001 for treatment and *P* = 0.024 for group effects/12-mm beam: *P* < 0.0001 for treatment and *P* = 0.0931 for group effects/6-mm beam: *P* < 0.0001 for treatment and *P* = 0.0062 for group effects/unsuccessful trials: *P* < 0.0001 for treatment and *P* = 0.0104 for group effects). Summary data are means ± SEM. Extended statistical data are provided in table S1. n.s., *P* > 0.05; *P < 0.05; **P < 0.01; ***P < 0.001; and ****P < 0.0001.

As the reduction in M4 receptor postsynaptic function (Figs. 1F and 2, B and E) was greater than the decrease observed in M4 receptor expression (Fig. 2C), additional changes in signaling cascades downstream of M4 receptor may also occur following DA loss. To examine these potential deficits, we next attempted to boost M4 receptor signaling by generating mice lacking regulator of G protein signaling protein (RGS4) selectively in dMSNs (Fig. 4, A and B). The timing and extent of G protein–coupled receptors (GPCRs) signaling is controlled by several classes of guanosine triphosphatase–activating proteins, including RGS proteins, which accelerate guanosine 5′-triphosphate (GTP) hydrolysis and facilitate termination of G protein signaling (*39*). In the striatum, RGS4 is highly expressed, regulates G_q_ and G_i/o_ (*40*, *41*), and has been implicated in the regulation of cholinergic and dopaminergic signaling, being relevant in several neurological disorders, including PD (*13*, *29*, *42*–*44*). As RGS4 preferentially regulates signaling by G_i/o_-coupled GPCRs (*40*, *41*), such as M4 receptors, we reasoned that selective ablation of RGS4 may enhance M4 signaling and thus rescue the reduction in M4 transmission arising after DA depletion (Fig. 4, A and B). To test this, we generated RGS4 conditional knockout (RGS4 cKO) (*RGS4^fl/fl^; D1-Cre^+/−^*) mice by crossing RGS4-floxed (*45*–*47*) and D1-Cre mice (Fig. 4A). Knockout of RGS4 increased the amplitude of electrically evoked M4-IPSCs (Fig. 4C and fig. S4A) and prolonged the kinetics of activation and deactivation compared to littermate controls (*RGS4^wt/wt^; D1-Cre^+/−^*) (fig. S4A), demonstrating that RGS4 regulates M4 signaling in dMSNs. Maximum outward currents evoked by Oxo-M (10 μM) were also increased, while M4 receptor sensitivity remained unaffected (Fig. 4D and fig. S4B). Knocking out RGS4 from dMSNs had no effect on ChI excitability (fig. S4C).

To determine whether prolonging M4 signaling by selective removal of RGS4 might limit the reduction in cholinergic transmission following DA depletion, we again performed a complete DA lesion using 6-OHDA (Fig. 4A). In line with our previous findings, the amplitude of M4-IPSCs in control mice was decreased in response to DA loss (Fig. 4C). The impairment in cholinergic transmission was restored in 6-OHDA–treated mice selectively lacking RGS4 in dMSNs, such that despite DA depletion, the amplitude of evoked M4-IPSCs was undistinguishable from saline-treated controls (Fig. 4C). This rescue of M4 transmission in parkinsonian mice was observed across a range of electrical stimulation intensities (fig. S4D) and also when examining maximal postsynaptic M4-mediated outward currents evoked by bath application of Oxo-M (Fig. 4D). The extent of DA depletion was complete for both 6-OHDA–lesioned conditions, as no differences were found in the forelimb use asymmetry between control and RGS4 cKO mice (fig. S4E). Thus, selective ablation of RGS4 in dMSNs was sufficient to rescue the impairment in M4-mediated cholinergic transmission following DA lesion, restoring levels of transmission back to those of control.

We next examined the impact of selective RGS4 removal in dMSNs on 6-OHDA–induced motor impairment. While global knockout of RGS4 has been reported to either decrease (*43*) or increase (*48*) parkinsonian motor deficits, cell type–specific effects of RGS4 ablation from dMSNs have not been addressed. Rotarod performance and open field locomotor activity were slightly impaired in saline-treated RGS4 cKO mice (Fig. 4, E and F). As M4 transmission was enhanced to similar extent by either overexpressing M4 receptor or prolonging its intracellular signaling (Figs. 3C and 4C), the additional impairment in basal locomotion might be due to the bilateral nature of this strategy or to alterations in RGS4-mediated regulation of other G_i/o_- or G_q_-coupled GPCRs in dMSNs or other D1-expressing cells. Normalizing activity to account for these basal impairments, however, revealed a rescue of both locomotion (Fig. 4E) and latency to fall from the rotarod (Fig. 4F), following RGS4 ablation in 6-OHDA–treated parkinsonian mice. Conditional knockout of RGS4 in dMSNs had no effect on basal performance in the balance beam test but again led to a partial rescue of 6-OHDA–induced motor deficits, including an improvement in the latency to cross the beam (Fig. 4G), a decrease in the number of foot slips (fig. S4G), and a reduction in the percentage of unsuccessful trials due to hesitation to start movement (Fig. 4G). Thus, in agreement with the previous findings, these results suggest that the reduction of postsynaptic M4 function in response to DA lesion may contribute to certain aspects of parkinsonian motor dysfunction.

### Reduced M4-cholinergic transmission following DA loss contributes to levodopa-induced dyskinetic behavior

**Fig. 5.**
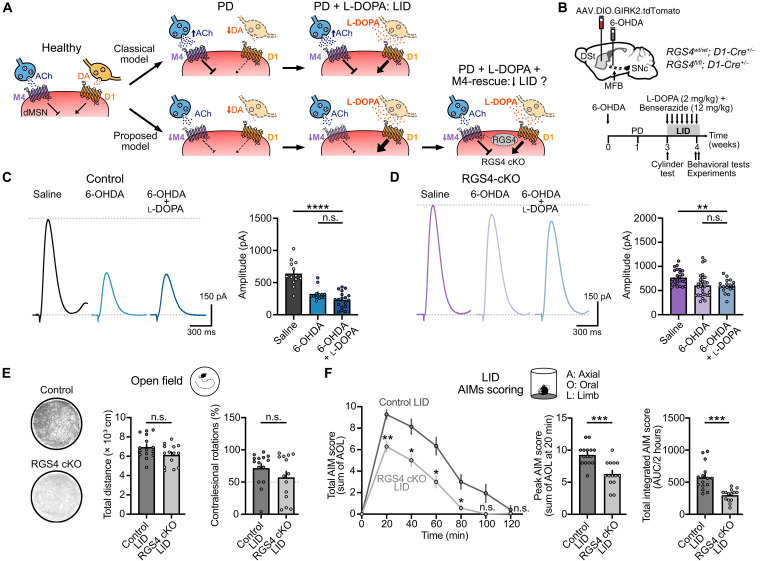
Restoration of M4 transmission alleviates l-DOPA–induced dyskinetic behavior. (**A**) Cartoon schematics showing the expected changes in the opposite regulation exerted by D1- and M4 receptor signaling pathways in dMSNs in PD and LID according to the classical model (top) and the proposed model (bottom). (**B**) Schematics of AAV9.hSyn.DIO.tdTomato.T2A.GIRK2 and 6-OHDA injections into DSt and MFB respectively in control and RGS4 cKO in dMSNs mice (top). Timeline for LID model development and experiments (bottom). (**C**) Representative traces and quantification of electrically evoked M4-IPSCs in control mice (saline: *n* = 13, *N* = 7; 6-OHDA: *n* = 13, *N* = 6; 6-OHDA + l-DOPA: *n* = 18, *N* = 5; *P* < 0.0001; Kruskal-Wallis and Dunn’s post hoc tests). Data from saline and 6-OHDA conditions were taken from Fig. 4C. (**D**) Representative traces and quantification of electrically evoked M4-IPSCs in RGS4 cKO mice (saline: *n* = 24, *N* = 11; 6-OHDA: *n* = 29, *N* = 10; 6-OHDA + l-DOPA: *n* = 18, *N* = 4; *P* < 0.0001; Kruskal-Wallis and Dunn’s post hoc tests). Data from saline and 6-OHDA conditions were taken from Fig. 4C. (**E**) Example traces of locomotor activity and quantification of total distance (left) and percentage of contralesional rotations in the open field (right) (control LID: *N* = 15; RGS4 cKO LID: *N* = 14; *P* = 0.0603, unpaired *t* test for total distance; *P* = 0.5323, Mann-Whitney for rotations). (**F**) Plot showing total AIM score as a function of time (left) (control LID: *N* = 15; RGS4 cKO LID: *N* = 14; *P* < 0.0001 for time and subject effects and *P* = 0.0001 for group effect; two-way RM ANOVA and Šídák’s post hoc test). Bar graphs show the summary data for AIM score peak at 20 min (center) and the integrated AIM score for the total dyskinetic period (right) (*P* = 0.0001; unpaired *t* tests). Summary data are means ± SEM. Extended statistical data are provided in table S1. n.s., *P* > 0.05; *P < 0.05; **P < 0.01; ***P < 0.001; and ****P < 0.0001.

As PD progresses, prolonged l-DOPA administration can lead to the development of levodopa-induced dyskinetic involuntary movements (*30*). Although LID pathogenesis remains incompletely understood, the combination of dopaminergic denervation and chronic pulsatile stimulation of DA receptors is thought to drive an enhancement of the direct pathway due to activation of supersensitive D1 receptors and their downstream signaling cascade (Fig. 5A) (*49*–*53*). In dMSNs, G_i/o_-coupled M4 receptors have been shown to directly oppose G_olf/s_ -coupled D1 receptor signaling (Fig. 5A) (*26*–*29*). Historically, as PD has been assumed as a hypercholinergic state (*3*, *4*, *6*), the combination of low DA and high ACh tone has been believed to result in a strong inhibition of the direct pathway and suppression of movement through reduced D1 signaling and enhanced M4 signaling. l-DOPA treatment is thought to rescue the reduced D1 signaling, with the heightened D1 receptor supersensitivity potentially balanced by an enhanced M4 signaling. However, as our findings revealed a reduction in M4 function in response to DA loss, a stronger imbalance between D1 receptor and M4 receptor signaling might then accelerate or aggravate the progression to LID once l-DOPA treatment is initiated (Fig. 5A). If this is the case, restoring M4 function in dMSNs may be expected to reduce the severity and limit the development of LID (Fig. 5A). To test this, we examined the effect of selectively ablating RGS4 from dMSNs in 6-OHDA–lesioned mice treated for 7 to 8 days with daily intraperitoneal injections of l-DOPA (2 mg/kg) plus benserazide (12 mg/kg) (Fig. 5B), a treatment paradigm that elicited both therapeutic antiparkinsonian effects (Fig. 5E) and AIMs typical of LID (Fig. 5F) in this PD mouse model. A similar extent of DA depletion for all parkinsonian mice was confirmed before starting l-DOPA injections by examining forelimb use asymmetry (fig. S5A).

We initially began by examining control mice to see if the 6-OHDA–induced reduction in M4 function could be rescued by prolonged l-DOPA administration. The amplitude of M4-IPSCs from GIRK2^+^ dMSNs from control 6-OHDA–lesioned mice (*RGS4^wt/wt^; D1-Cre^+/−^*) treated with l-DOPA, however, was still significantly reduced compared to saline animals and no different than parkinsonian mice not treated with l-DOPA (Fig. 5C). This suggests that once initiated, the decrease in M4 function becomes sustained and resistant to changes that replace striatal DA. Similarly, repeating this experiment in RGS4 cKO mice (*RGS4^fl/fl^; D1-Cre^+/−^*) showed that while removal of RGS4 from dMSNs still prevented a loss of M4 function following DA lesion (fig. S5B), no further potentiation was seen after l-DOPA treatment (Fig. 5D). Thus, while the decrease in M4 function can be driven by DA loss, exogenously raising DA levels after lesion with l-DOPA was unable to revert the decreased cholinergic transmission.

We next took advantage of the fact that l-DOPA failed to alter M4-mediated cholinergic transmission to evaluate the impact of restoring M4 function on the antiparkinsonian and dyskinetic effects induced by l-DOPA, again hypothesizing that restoring direct pathway M4 function would be expected to limit the severity and development of LID by balancing the enhanced D1 signaling (Fig. 5A). The antiparkinsonian or therapeutic effects of l-DOPA administration were assessed by analyzing locomotor activity and frequency of rotations in an open field arena during a 30-min observation period in the “on” phase of l-DOPA treatment (Fig/ 5E). Knockout of RGS4 did not prevent l-DOPA from increasing total distance traveled, velocity or contralesional turning, the latter of which was restored to balanced levels expected for healthy animals (Fig. 5E, fig. S5C, and table S1). Thus, restoration of M4-mediated cholinergic transmission in mice lacking RGS4 did not compromise the therapeutic antiparkinsonian effects of l-DOPA treatment.

To directly quantify l-DOPA-induced dyskinetic behavior, AIMs for each group were determined by measuring abnormal axial, oral, and limb (AOL) movements (Fig. 5F) (*54*). While l-DOPA–treated control mice rapidly developed dyskinesia, with a total dyskinetic event duration of 120 min, RGS4-lacking mice progressed to dyskinesia at slower rate, terminating the dyskinetic period faster, and with an attenuation of the AIMs peak (Fig. 5F). Moreover, the overall total AIM score was significantly reduced across the testing period as can be seen by comparing the integrated AIM score or area under the curve (AUC) for the total dyskinetic period (Fig. 5F). Overall, these results indicate that the reduction in direct pathway M4-mediated cholinergic transmission following DA loss accelerates and aggravates LID, so that restoration of M4 function ameliorates the dyskinetic behavior without compromising the antiparkinsonian effects of l-DOPA treatment.

## DISCUSSION

While support for the role of altered cholinergic signaling in PD has come from early ACh dialysis studies (*5*) and clinical observations where broad spectrum antimuscarinic drugs partially ameliorate PD motor symptoms (*55*, *56*), directly examining changes in cholinergic signaling in dMSNs following DA depletion has remained unexplored. Here, we examined this issue and found that, opposite of expected, cholinergic transmission was reduced in response to DA loss. This resulted from impaired postsynaptic M4 function and involved both down-regulation of receptors and weakened downstream signaling because either overexpressing M4 receptors or enhancing signaling by selective knockout of RGS4 was sufficient to restore transmission. Reverting impaired M4 transmission led to a partial alleviation of parkinsonian balance and coordination motor deficits, as well as LID dyskinetic behavior, revealing select prokinetic effects in addition to the classical antikinetic role ascribed to cholinergic signaling. Thus, reduced M4-mediated cholinergic signaling in dMSNs constitutes a previously unrealized alteration that differentially contributes to PD symptomatology and LID progression.

The reduction in direct pathway postsynaptic M4 function may constitute an adaptative or compensatory change aimed to counteract diminished D1 receptor stimulation in dMSNs following DA loss. The decrease in M4 expression at the protein level supports past work showing reduced striatal M4-mRNA levels (*57*, *58*) and M2/M4 radioligand binding (*59*) in parkinsonian models [yet, see (*7*)]. As M4 receptor surface levels are decreased in MSNs following prolonged agonist stimulation or changes in cyclic adenosine 3′,5′-monophosphate (cAMP) levels, the reduction in M4 expression might be driven by sustained alterations in ACh and/or DA tone (*60*–*62*). Besides a decrease in M4 expression, additional impairments in downstream signaling cascades also likely occur following DA loss. This could be seen in experiments where we overexpressed M4 receptors. While overexpression similarly increased the total receptor levels in saline and 6-OHDA–treated mice, M4 function still remained reduced in lesioned animals. One potential downstream signaling candidate may be RGS4, as we found that it regulates M4 signaling in dMSNs and that its ablation is sufficient to restore the decreased M4 transmission in parkinsonian mice. As RGS4 transcription appears to be bidirectionally regulated by DA receptor–mediated cAMP signaling, being up-regulated when cAMP levels are low or unchanged, and downregulated when cAMP increases (*63*–*65*), RGS4 mRNA levels can be dynamically and differentially altered in response to acute or prolonged DA depletion (*13*, *42*, *44*). In addition to dMSNs, M4 receptors are also expressed in ChIs and glutamatergic projections from cortex and thalamus (*20*, *21*). Although M4 autoreceptors on ChIs are reduced following DA lesion (*13*), M4 receptor function on cortical terminals was unaltered (*7*). As M4 expression and RGS4 mRNA levels may be distinctively regulated across cell types and/or projecting fibers to the striatum (*13*, *60*), future work is needed to determine whether the adaptation in direct pathway M4 function and its underlying mechanisms are cell type specific or common to all striatal elements. Note that our study only focused on postsynaptic M4 receptors at the somatodendritic compartment of dMSNs in the striatum. However, as M4-mediated inhibition of D1 signaling and GABA release also occurs at dMSNs terminals in the substantia nigra pars reticulata (SNr) and involves extrastriatal sources of ACh (*26*), it remains to be tested whether similar adaptive changes in M4 signaling occur throughout all dMSN compartments.

As M4 receptors are G_i/o_-coupled, activation by ACh is classically thought to lead to inhibition of the direct pathway and movement suppression, playing an antikinetic role (*23*–*26*). In partial agreement with this, we found that increasing M4 expression or signaling in dMSNs in control mice led to an impaired basal performance in some, but not all, motor tasks. The surprise from our work was that following DA depletion, reverting the reduction in direct pathway M4 transmission back to normal levels led to a partial alleviation rather than a worsening of motor behavior in parkinsonian mice. This suggests a prokinetic effect of ACh signaling for select aspects of movement, especially in balance and coordination. Patients with PD symptoms associated with these features—such as postural instability, gait dysfunction, freezing of gait, and falls—are usually unresponsive to DA replacement therapy (*38*, *66*, *67*) and have been linked to nondopaminergic systems, particularly to cholinergic dysfunction in the striatum and downstream basal ganglia circuits (*68*–*70*). Along the lines of the antikinetic function ascribed to M4 receptors, restoring M4 transmission back to normal levels by selective ablation of RGS4 also limited the development of LID dyskinetic behavior but without compromising the therapeutic antiparkinsonian effects of l-DOPA. A previous study found that pharmacologically enhancing M4 function with positive allosteric modulators (M4-PAMs) partially alleviates AIMs in a more advanced model of LID (*29*). Our work showing that there is a decrease in M4 function in dMSNs in response to DA loss, which could not be reversed by l-DOPA, suggests that this is a factor that accelerates or potentially compounds the progression toward LID. As this contribution might be more relevant at initial stages of dyskinesia, we focused on a more moderate paradigm of l-DOPA administration. Regarding the cholinergic rescue strategy, we chose to examine the selective ablation of RGS4 in dMSNs over the overexpression of M4 receptor for LID experiments due to the higher reproducibility achieved by a genetic versus a viral-mediated approach, and the fact that while RGS4 cKO may alter other GPCR-signaling [e.g., G_q_-coupled mGluR5 (*29*)], it has biased preference for G_i/o_-coupled receptors (*40*, *41*), suggesting that M4 signaling regulation may be the primary effector in dMSNs. While future studies are needed to directly examine LID in mice with enhanced M4 expression, we would expect a similar amelioration of the dyskinetic behavior by virally overexpressing M4 receptor levels because both approaches had comparable overall impacts on cholinergic transmission and parkinsonian motor impairment.

At first glance, the fact that reduced M4 transmission contributes to both parkinsonian and LID motor dysfunction may seem counterintuitive. However, our findings highlight how ACh and DA interactions may be much more complex and coordinated than the solely antagonistic roles traditionally assumed. It has been shown that ACh does not only oppose to DA signaling, but that both neuromodulators may act in concert to regulate different aspects of movement (*71*, *72*), so that ChIs seem central for orchestrating motor functions involving balance and coordination (*73*). Here, we revealed that direct pathway M4-mediated modulation of dMSNs can also exert a modest prokinetic effect in addition to its commonly ascribed antikinetic role. Thus, while the antikinetic effect of M4 signaling was observed in control and LID conditions when DA is present, its prokinetic actions appeared only when striatal DA is depleted. This suggests that the presence or absence of DA may determine the dominant effect of cholinergic signaling through M4 receptors. Previous reports have shown that M4 receptors in dMSNs can mediate inhibition or facilitation via Gα_i/o_ protein (*26*–*29*) or cyclin Cdk5 signaling pathways, respectively (*36*), which are thought to be differentially engaged depending on the temporal sequence of D1- and M4 receptors activation (*35*, *36*). Moreover, this alternate signaling pathway for M4 receptors seems to be reversed following DA depletion (*36*). It might also be possible that the different kinetic effects mediated by M4 receptors are the result of signaling at different subcellular compartments in dMSNs and/or by engaging different circuits. Previous work has shown that M4-mediated inhibition at dMSNs terminals at the SNr is associated with an antikinetic role (*26*), while M4-mediated facilitation has been described at the perisomatic region of dMSNs (*35*, *36*), which may suggest that the prokinetic effect originates at striatal level. However, again, future studies will be required to dissect the specific and potentially different signaling mechanisms, subcellular compartments, and/or activated circuitries underlying these diverse effects in movement.

The rationale for the use of antimuscarinic agents to treat motor symptoms in PD arose with the classical view predicting an enhanced M4 transmission following DA loss and exclusive antikinetic effects for ACh. If the compensatory or adaptive reduction in M4 signaling observed in our preclinical model remains true for the human disease, muscarinic antagonists should be more effective at early stages, when DA levels are not completely depleted yet. In this regard, selective M4 antagonists might be preferred to limit the side effects ascribed to other central and peripheral muscarinic receptors (*74*). In more advanced stages of dopaminergic degeneration, when M4 signaling would be already down-regulated, the efficacy of antimuscarinic agents may be more limited, as it has been reported for bradykinesia and rigidity (*55*, *56*), and motor features such as balance and coordination, partially relying on ACh prokinetic effects, might even be worsen. DA replacement still remains as the most effective therapy for core parkinsonian deficits in these advanced stages, and our results suggest that further inhibiting M4 transmission by antimuscarinic antagonists concomitantly to l-DOPA treatment would aggravate LID (*23*, *75*). Therefore, strategies aimed to restore or moderately enhance M4 signaling, such as the ones described here, might be preferred as an adjunct to l-DOPA and perhaps other DA-replacement medications. Futhermore, they may also be beneficial for DA-unresponsive deficits, which are often linked to balance and coordination. In line with our results, pharmacological approaches such as M4-PAMs (*29*, *76*) or RGS4 inhibitors (*77*, *78*) have exhibited promising results in preclinical models of both PD and LID.

One potential caveat that is important to note is that as the present study focused on postsynaptic M4 receptor changes in vitro, it remains unknown to what extent M4 receptor signaling would be altered in vivo, where additional alterations in ChI excitability/activity or ACh release may also occur. This is especially relevant because differing results—including increases (*5*, *8*, *13*, *16*, *18*, *19*, *79*), decreases (*12*, *14*, *17*), or even lack of changes (*11*, *15*, *80*)—have been found in different animal models of PD. It has also been suggested that the relative balance between ACh and DA signaling is more determinant for striatal circuitry (*17*), and in that scenario, even a reduced cholinergic transmission would still outweigh the almost nonexistent dopaminergic signaling in advanced PD. Thus, the predicted hypercholinergic nature of this disease still remains unclear, and further investigation will be required to determine whether it coexists with or indirectly contributes to the decreased postsynaptic M4 receptor function.

In conclusion, our findings reveal an unexpected reduction in striatal M4-mediated cholinergic transmission in a parkinsonian model, which may constitute a previously unnoticed adaptive change in response to DA loss that differentially contributes to both PD and LID motor circuit dysfunction. These results underscore the promising therapeutic potential of targeting striatal M4-mediated cholinergic transmission dynamically at the different stages of dopaminergic degeneration in the disease and while undergoing DA-replacement treatment, encouraging further preclinical and clinical research on M4 receptors.

## MATERIALS AND METHODS

### Animals

All animal experiments were performed in agreement with the protocols approved by Institutional Animal Care and Use Committee at the University of Colorado School of Medicine (approval number: 0155). Animals were group-housed in a temperature- and humidity-controlled environment on a 12-hour light/12-hour dark cycle, with water and food available ad libitum, and experiments were conducted during the light phase. Both adult male and female 10- to 11-week-old mice were used including: wild-type C57BL/6J mice (the Jackson Laboratory, RRID:IMSR_JAX:000664), Adora2a-Cre heterozygote mice (RRID:MMRRC_036158-UCD), Drd1-Cre heterozygote mice (MMRRC, RRID:MMRRC_034258-UCD), and RGS4 cKO (*RGS4^fl/fl^*:: *Drd1-Cre^+/wt^*; RGS4 cKO) in D1-MSNs and littermate controls (*RGS4^wt/wt^:: Drd1-Cre^+/wt^*), which were generated by crossing Drd1-Cre mice with RGS4^fl/fl^ mice provided by V. Zachariou (*45*–*47*). MitoPark (*Tfam^fl/fl::^ DAT-Cre^+/−^*) and littermate control mice (*Tfam^fl/fl^:: DAT-Cre^−/−^*), obtained by crossing DAT-IRES-Cre mice (the Jackson Laboratory, RRID: IMSR_JAX: 006660) with Tfam^fl/fl^ mice (the Jackson Laboratory, RRID: IMSR_JAX:026123), were used at 6 to 7 months old.

### Stereotaxic surgery

Mice (7 to 8 weeks old or 5 to 6 months old for MitoPark and littermate controls) were anesthetized with isoflurane, mounted in a stereotaxic frame (Kopf Instruments), and kept under constant 2% isoflurane anesthesia. AAV viruses were injected using a Nanoject III (Drummond Scientific) at 2 nl/s into the center of the DSt at the following coordinates relative to bregma (in mm): anterior-posterior (AP), +0.9; medial-lateral (ML), ±1.85; dorso-ventral (DV), −2.9. The needle was kept in the target site for 5 min to allow diffusion. For experiments involving only cell type–specific GIRK2 expression, 400 nl of AAV9.hSyn.DIO.tdTomato.T2A.GIRK2 (University of Pennsylvania Viral Core, RRID: SCR_022432, V5688R) was injected, while AAV9.hSyn.tdTomato.T2A.mGIRK2 (University of Pennsylvania Viral Core, RRID: SCR_022432, V6321R) was injected for nonselective expression in the DSt. For experiments where GIRK2 was selectively coexpressed with M4 receptor or eYFP in dMSNs, 300 nl of AAV9.hSyn.DIO.tdTomato.T2A.GIRK2 was co-injected with 300 nl of AAV9-EF1a-DIO-mChrM4-P2A-eGFP-WPRE-SV40pA (Virovek, provided by Y. Zhu and J. Javitch) or AAV5.EF1a.DIO.EYFP (Addgene, 27056, RRID:Addgene_27056), respectively. For experiments where only fluorescent reporters were selectively expressed in dMSNs, 400 nl of either AAV5.EF1a.DIO.EYFP or AAV5-Flex-CAG-tdTomato (Addgene, 28306, RRID:Addgene_28306) was used.

### Unilateral 6-OHDA mouse model of PD

A high dose (4 μg/μl) of 6-OHDA (6-OHDA hydrobromide; Sigma-Aldrich, 162957) dissolved in sterile saline was injected (1 μl) unilaterally into the MFB during stereotaxic surgery at the following coordinates relative to bregma (in mm): AP, −1.2; ML, +1.3; DV, −4.75. Mice were pretreated with desipramine (25 mg/kg) and pargyline (5 mg/kg) dissolved in sterile saline 30 min before 6-OHDA injections. To minimize mortality, special care was conducted during at least 1 week after surgery. Briefly, mice remained in a heat pad and received daily sterile saline intraperitoneal injections, food pellets soaked in water, and nutritionally fortified water gel (DietGel Recovery, ClearH20).

### LID mouse model

Three weeks after 6-OHDA injections, mice were examined by the cylinder test to verify complete DA depletion and then received daily intraperitoneal injections of l-DOPA (2 mg/kg; Sigma-Aldrich, D9628) plus benserazide hydrochloride (12 mg/kg; Sigma-Aldrich, B7283) dissolved in sterile saline for 7 to 8 days. Dyskinetic movement was formally scored at the end of the treatment. Brain slices were prepared 30 min to 1 hour after the last l-DOPA administration, when mice were still in the dyskinetic period.

### Slice preparation

Mice were anesthetized with isoflurane and transcardially perfused with ice-cold sucrose cutting solution containing 75 mM NaCl, 2.5 mM KCl, 6 mM MgCl_2_, 0.1 mM CaCl_2_, 1.2 mM NaH_2_PO_4_, 25 mM NaHCO_3_, 2.5 mM d-glucose, and 50 mM sucrose, bubbled with 95% O_2_ and 5% CO_2_. Coronal striatal slices (240 μm) were cut in the same ice-cold sucrose cutting solution. Slices were then incubated for at least 45 min at 32°C in artificial cerebrospinal fluid (ACSF) containing 126 mM NaCl, 2.5 mM KCl, 1.2 mM MgCl_2_, 2.5 mM CaCl_2_, 1.2 mM NaH_2_PO_4_, 21.4 mM NaHCO_3_, and 11.1 mM d-glucose, bubbled with 95% O_2_ and 5% CO_2_. A total of 10 μM MK-801 was added to prevent excitotoxicity. After incubation, slices were transferred into a recording chamber and constantly perfused with ACSF warmed to 32° ± 2°C at a rate of 2 ml/min. Neurons were visualized using a BX51WI microscope (Olympus) with an infrared light-emitting diode (LED), while green and blue LEDs were used for visualizing fluorescence (Thorlabs).

### Electrophysiology

All recordings were conducted in the DLS using Axopatch 200B amplifiers (Molecular Devices). Patch pipettes (1.5 to 2 megohm) (World Precision Instruments) were made using a pipette puller (Narishige, PC-10). Pipettes for whole-cell recordings from MSNs contained 135 mM d-gluconate (K), 10 mM Hepes (K), 0.1 mM CaCl_2_, 2 mM MgCl_2_, and 10 mM 1,2-bis(2-aminophenoxy)ethane-*N*,*N*,*N*′,*N*′-tetraacetic acid (BAPTA)–tetra potassium, with adenosine 5′-triphosphate (ATP) (1 mg/ml), guanosine 5′-triphosphate (GTP) (0.1 mg/ml), and phosphocreatine (1.5 mg/ml) (pH 7.35, 275 mOsm). MSNs were held at −60 mV. To reduce the variability of GIRK2 outward currents between cells and animals, all recordings from GIRK2^+^ MSNs were conducted in regions showing robust reporter fluorescence, and where indicated, ACh release was triggered by electrical stimulation using a monopolar glass stimulating electrode filled with ACSF, positioned 200 μm away from the recorded cell. No series resistance compensation was applied, and cells were discarded if series resistance was ≥15 megohm. For ChI recordings, the internal solution contained 135 mM d-gluconate (K), 10 mM Hepes (K), 0.1 mM CaCl_2_, 2 mM MgCl_2_, and 0.1 mM EGTA, with ATP (1 mg/ml), GTP (0.1 mg/ml), and phosphocreatine (1.5 mg/ml) (pH 7.35, 275 mOsm). All putative ChIs were identified by their large size and the presence of a hyperpolarization-activated inward current (H-current) with a hyperpolarization protocol (30 mV, 5 s). Recordings were acquired with Axograph 1.76 (Axograph Scientific; RRID SCR_014284) at 10 kHz and filtered to 2 kHz or with LabChart (ADInstruments; RRID:SCR_017551) at 1 kHz. Unless otherwise noted, all drugs were bath applied, and recordings were performed in ACSF containing 10 μM DNQX, 100 μM picrotoxin, 300 nM CGP 55845, 1 μM SCH 23390, 1 μM DHβE, and 200 nM sulpiride to isolate muscarinic cholinergic transmission.

### Electrophysiology and two-photon imaging of Ca^+2^ transients

Whole-cell current-clamp recordings of dMSNs in the DLS were conducted using electrodes filled with the 135 mM d-gluconate (K), 10 mM Hepes (K), 4 mM MgCl_2_, ATP (1 mg/ml), GTP (0.1 mg/ml), phosphocreatine (1.5 mg/ml), 10 μM Alexa Fluor 594 (Invitrogen, A10438), and 100 μM Fluo-5F (AAT Bioquest, 20562) (pH 7.35, 275 mOsm). Single bAPs were elicited by somatic depolarizing current injection (200 to 400 pA, 100 ms), and Ca^+2^ transients were measured at the shaft or spines of proximal dendrites (40 to 60 μm from soma) by two-photon imaging before and after bath application of a saturating concentration of the muscarinic agonist Oxo-M (10 μM). Two-photon calcium imaging was performed using a two-photon laser scanning microscopy system, custom-built on a BX51WI microscope (Olympus). A Ti:Sapphire laser (Chameleon Ultra I; Coherent) was tuned to emit pulsed excitation at 810 nm and scanned using a pair of X-Y galvanometer mirrors (6215, Cambridge Technology). Emitted fluorescence was collected through a water-immersion objective (60×, Olympus), a dichroic mirror (T700LPXXR, Chroma), and filters (ET680sp and ET525/50 m-2P, Chroma) and was detected using a GaAsP photomultiplier tube (PMT; H10770PA-40, Hamamatsu). A current preamplifier (sensitivity, 100 nA/V; SR570, Stanford Research Systems) was used to convert the output to voltage, which was then digitized by a data acquisition card (PCI-6110, National Instruments). Peak fluorescent changes of the calcium indicator Fluo-5F were measured using two-photon spot nonraster scanning photometry with a custom software (Toronado; https://github.com/StrowbridgeLab/Toronado-Laser-Scanning) as previously described (*81*). The laser was repeatedly scanned across a small circular path (150 nm in diameter) at the selected region of interest, and fluorescence was continuously collected from that spot. The PMT signal was converted by the preamplifier and further filtered to 500 Hz with the gain increased twofold (FLA-01, Cygnus Technologies). Then, the signal was acquired using a data acquisition device (ITC-18, HEKA Instruments) and recorded and analyzed using Axograph X (Axograph Scientific, RRID SCR_014284). Three to five dendritic spots in the same optical plane were imaged and averaged per cell. In addition to glutamate, DA, GABA, and nicotinic receptor antagonists, 500 nM VU-0255035 was added to bath ACSF to selectively block M1 receptors.

### Immunohistochemistry and fluorescence imaging

Mice were anesthetized using isoflurane and perfused transcardially with cold phosphate-buffered saline (PBS) followed by cold 4% paraformaldehyde in PBS (pH 7.4). Brains were post-fixed in 4% paraformaldehyde at 4°C for additional 2 hours, equilibrated in 30% sucrose solution for 2 days, and rapidly frozen in embedding freezing medium (Thermo Fisher Scientific). DSt and/or midbrain coronal slices of 30 μm in thickness were obtained using a Leica CM1950 cryostat (Leica Microsystems).

For TH immunohistochemistry, sections were mounted on slides and blocked in 5% normal donkey serum in PBS-T (0.3% Triton X-100) for 1 hour at room temperature (RT). Slides were then washed in PBS and incubated with rabbit anti-TH primary antibody (1:200; Millipore AB152, RRID: AB_390204) overnight at 4°C. After PBS washes, slides were incubated with donkey anti-rabbit Alexa Fluor 488 secondary antibody (1:500; Life Technologies A21206, RRID: AB_2535792) for 1 hour at RT and washed afterward with PBS.

For M4 receptor immunohistochemistry, sections were mounted on slides and stained using a monoclonal mouse anti-muscarinic receptor M4/CHRM4 primary antibody (1:500; Abcam, ab77956, RRID: AB_1566454), with blocking reagents and secondary anti-mouse biotinylated antibody from the M.O.M. Immunodetection Kit according to the manufacturer’s directions (Vector Laboratories, BMK-2202, RRID:AB_2336833). Sections were then incubated with streptavidin Alexa Fluor 594 conjugate (1:1000; Invitrogen, S32356) for 30 min at RT and washed afterward with PBS.

Following immunostaining, sections were finally mounted with an anti-fading mounting media. When only visualizing fluorescence reporters, sections were mounted right after being washed in PBS without additional immunostaining. Fluorescent images were acquired using a slide scanner microscope (VS120, Olympus) and processed in Fiji (ImageJ, RRID:SCR_002285).

### Western blot

DSt tissue for Western blot analysis was collected from saline and 6-OHDA–injected hemispheres and then further dissected with a sagittal section along the midline to separate dorsomedial and dorsolateral regions. The samples were homogenized and denatured with STE buffer [10 mM tris-Cl (pH 7.5), 1 mM EDTA pH 8.0, and 1% SDS] at 100°C for 5 min. Protein concentrations were quantified by Pierce BCA Protein Assay (Thermo Fisher Scientific, 23225-7). Equivalent amounts of protein were subjected to SDS–polyacrylamide gel electrophoresis on 10% polyacrylamide gels and then transferred to methanol activated polyvinylidene difluoride membranes (PerkinElmer). Blots were blocked with 5% milk in tris buffered saline with tween (0.1% Tween) (TBS-T) for 1 hour at RT and then immunolabeled with rabbit primary antibodies, anti-GIRK2 (1:500; Alomone labs, APC-006, RRID: AB_2040115) and anti-actin (1:2000; Cell Signaling Technology, 4970S, RRID: AB_2223172) overnight at 4°C in blocking buffer. Blots were then probed with horseradish peroxidase–conjugated secondary antibody (1:3000; GE Healthcare, NA934, RRID: AB_772206) for 1 hour at RT. Proteins were detected using a chemiluniscent substrate (SuperSignal West Pico PLUS Chemiluniscent Substrate, Thermo Fisher Scientific, 34577) and visualized in FluorChem SP (Alpha Innotech). Densitometry analysis was performed in Fiji (ImageJ, RRID:SCR_002285).

### Chemicals

Picrotoxin (1128), (+)-MK-801 maleate (0924), DNQX (0189), DHβE hydrobromide (2349), SCH 23390 hydrochloride (0925), (S)-(−)-sulpiride (0895), Ach chloride (2809), oxotremorine-M (1067), VU 0255035 (3727), tropicamide (0909), ambenonium dichloride (0388), and scopolamine hydrobromide (1414) were obtained from Tocris Bioscience. EGTA (E4378), desipramine hydrochloride (D3900), 6-OHDA hydrobromide (162957), l-DOPA (D9628), and benserazide hydrochloride (B7283) were from Sigma-Aldrich. CGP55845 hydrochloride (HB0960) was purchased from Hello Bio, BAPTA tetra potassium salt was obtained from Invitrogen (B1204), and pargyline hydrochloride was obtained from Abcam (ab141265).

### Motor behavioral assessment

Prior to each behavioral test, mice were habituated to the testing room for 30 min. All behavioral tests were performed 3 weeks following stereotaxic injections. Video recordings were analyzed post hoc blindly.

### Cylinder test

Forelimb use asymmetry during exploratory activity was assessed by cylinder test. Individual mice were placed in a clear plastic cylinder (10.5 cm in diameter; 14.5 cm in height) with mirrors located behind for appropriate vision and were video recorded for 5 min for later post hoc scoring. No prior habituation was allowed before video recording, and only wall contacts executed with fully extended digits were scored. Data were expressed as a percentage of touches performed with the forelimb contralateral to the injected side with respect to the total paw use.

### Rotarod

Rotarod test was performed to assess motor balance and coordination using an accelerated protocol from 3 to 30 rpm in 5 min (Med Associates). Each mouse underwent three trials on the same day with a separation of 10 min between trials, without previous training. The latency to fall or time to reach maximum speed was recorded, and the data were expressed as the average of the two best trials.

### Balance beam

A balance beam test was conducted to assess motor balance and coordination by measuring mice ability to transverse a graded series of beams to reach a goal cage. Animals were trained for 1 day in the medium size square beam (12 mm), and the following day, that one and two additional beams (19 and 6 mm) were presented to the mice. Animals were video recorded while undergoing two trials per beam size, and the number of slips and time to cross the beam were registered, with a maximal score of 60 s (unsuccessful trial). The data were expressed as the average of the two trials for each beam size. The number of slips was only counted for successful trials (<60 s).

### Open field

Locomotion was assessed using the open field test. Each mouse was gently placed into a square arena (50 cm by 50 cm), and video was recorded for 10 min using an overhead camera. Tracking and post hoc analysis of total distance and velocity were conducted with Ethovision XT 17.5 (Noldus, RRID:SCR_004074).

For a subset of mice used for the LID model, open field test was assessed after 7 to 8 days of daily l-DOPA injections. Each mouse received the last l-DOPA intraperitoneal injection and was placed into a clear plastic cylinder (25.4 cm in diameter; 30.5 cm in height). After half an hour, mice were video recorded for another 30-min period using an overhead camera. Tracking and post hoc analysis of locomotion and rotations [threshold: 90°, minimum distance traveled: 2 cm, (*52*)] were automatically performed with Ethovision XT 17.5 (Noldus, RRID:SCR_004074).

### AIM score

Dyskinesia was assessed using the AIM score (*54*). Mice were individually placed in clear plastic cylinders (20 cm in diameter; 25.5 cm in height) following l-DOPA injection and abnormal AOL movements were video recoded for 1 min every 20 min for a total length of 2 hours. The AIM scale ranges from 0 to 4 for each body segment during a 1-min period: 0, normal movement; 1, abnormal movement for <50% of the time; 2, abnormal movement for >50% of the time; 3, abnormal movement for the entire period that can be interrupted by sensory stimuli; and 4, continuous abnormal movement, uninterruptible. The total AIM score is the sum of scores for AOL, being 12 the maximum score in 1 min. An integrated AIM score was calculated as the AUC in a plot of total AIM score versus time for the duration of the dyskinetic episode (2 hours).

### Statistical analysis

Statistical analyses were performed in Prism 10 (GraphPad 10.0.2, RRID:SCR_00306). All data are shown as means ± SEM. Datasets that passed the Shapiro-Wilk test for normality were analyzed using parametric tests; otherwise, nonparametric tests were applied. For comparison between two groups, the following two-tailed statistical tests were conducted as appropriate: unpaired or paired Student’s *t* test, Mann-Whitney *U* test, or Wilcoxon matched-pairs signed rank test. For comparisons between more than two groups, the following statistical tests were applied as appropriate: One-way analysis of variance (ANOVA), Kruskal-Wallis ANOVA, two-way ANOVA, or mixed-model ANOVA with Geisser-Greenhouse’s correction. Repeated measures (RM) version for those tests was performed for matched or paired data. When significant differences were found in ANOVA tests, post hoc multiple comparison tests were performed, including Dunnet’s, Dunn’s, Tukey’s, or Holm-Sidak’s tests. Concentration-response curves were adjusted by nonlinear regression (Hill coefficient = 1), and EC_50_ (median effective concentration) was assumed to be Gaussian distributed. Statistical significance was established as n.s. (not significant), *P* ≥ 0.05; *P < 0.05; **P < 0.01; ***P < 0.001; and ****P < 0.0001. “*n*” denotes number of cells, and “*N*” represents number of animals. *P* values, *n*, *N*, and specific statistical tests for each experiment and comparison are stated in the figure legends and table S1.

## References

[1] C. R. Gerfen, D. J. Surmeier, Modulation of striatal projection systems by dopamine. Annu. Rev. Neurosci. 34, 441–466 (2011). 21469956 10.1146/annurev-neuro-061010-113641 PMC3487690

[2] M. M. McGregor, A. B. Nelson, Circuit mechanisms of Parkinson’s disease. Neuron 101, 1042–1056 (2019). 30897356 10.1016/j.neuron.2019.03.004

[3] A. Barbeau, The pathogenesis of Parkinson’s disease: A new hypothesis. Can. Med. Assoc. J. 87, 802–807 (1962). 13966498 PMC1849683

[4] P. L. McGeer, J. E. Boulding, W. C. Gibson, R. G. Foulkes, Drug-induced extrapyramidal reactions. Treatment with diphenhydramine hydrochloride and dihydroxyphenylalanine. JAMA 177, 665–670 (1961). 13773933 10.1001/jama.1961.03040360001001

[5] P. DeBoer, E. D. Abercrombie, M. Heeringa, B. H. C. Westerink, Differential effect of systemic administration of bromocriptine and L-DOPA on the release of acetylcholine from striatum of intact and 6-OHDA-treated rats. Brain Res. 608, 198–203 (1993). 8495354 10.1016/0006-8993(93)91459-6

[6] T. Aosaki, M. Miura, T. Suzuki, K. Nishimura, M. Masuda, Acetylcholine-dopamine balance hypothesis in the striatum: An update. Geriatr. Gerontol. Int. 10, S148–S157 (2010). 20590830 10.1111/j.1447-0594.2010.00588.x

[7] G. Laverne, J. Pesce, A. Reynders, E. Combrisson, E. Gascon, C. Melon, L. Kerkerian-Le Goff, N. Maurice, C. Beurrier, Cholinergic interneuron inhibition potentiates corticostriatal transmission in direct medium spiny neurons and rescues motor learning in parkinsonism. Cell Rep. 40, 111034 (2022). 35793632 10.1016/j.celrep.2022.111034

[8] N. Maurice, M. Liberge, F. Jaouen, S. Ztaou, M. Hanini, J. Camon, K. Deisseroth, M. Amalric, L. Kerkerian-Le Goff, C. Beurrier, Striatal cholinergic interneurons control motor behavior and basal ganglia function in experimental Parkinsonism. Cell Rep. 13, 657–666 (2015). 26489458 10.1016/j.celrep.2015.09.034

[9] S. Ztaou, N. Maurice, J. Camon, G. Guiraudie-Capraz, L. Kerkerian-Le Goff, C. Beurrier, M. Liberge, M. Amalric, Involvement of striatal cholinergic interneurons and m1 and m4 muscarinic receptors in motor symptoms of Parkinson’s disease. J. Neurosci. 36, 9161–9172 (2016). 27581457 10.1523/JNEUROSCI.0873-16.2016 PMC6601910

[10] G. Kharkwal, K. Brami-Cherrier, J. E. Lizardi-Ortiz, A. B. Nelson, M. Ramos, D. Del Barrio, D. Sulzer, A. C. Kreitzer, E. Borrelli, Parkinsonism driven by antipsychotics originates from dopaminergic control of striatal cholinergic interneurons. Neuron 91, 67–78 (2016). 27387649 10.1016/j.neuron.2016.06.014 PMC4939839

[11] T. Aosaki, A. M. Graybiel, M. Kimura, Effect of the nigrostriatal dopamine system on acquired neural responses in the striatum of behaving monkeys. Science 265, 412–415 (1994). 8023166 10.1126/science.8023166

[12] S. J. Choi, T. C. Ma, Y. Ding, T. Cheung, N. Joshi, D. Sulzer, E. V. Mosharov, U. J. Kang, Alterations in the intrinsic properties of striatal cholinergic interneurons after dopamine lesion and chronic L-DOPA. eLife 9, e56920 (2020). 32687053 10.7554/eLife.56920 PMC7380940

[13] J. Ding, J. N. Guzman, T. Tkatch, S. Chen, J. A. Goldberg, P. J. Ebert, P. Levitt, C. J. Wilson, H. E. Hamm, D. J. Surmeier, RGS4-dependent attenuation of M4 autoreceptor function in striatal cholinergic interneurons following dopamine depletion. Nat. Neurosci. 9, 832–842 (2006). 16699510 10.1038/nn1700

[14] M. Herrera-Marschitz, J. Luthman, S. Ferré, Unilateral neonatal intracerebroventricular 6-hydroxydopamine administration in rats: II. Effects on extracellular monoamine, acetylcholine and adenosine levels monitored with in vivo microdialysis. Psychopharmacology 116, 451–456 (1994). 7701048 10.1007/BF02247477

[15] M. Herrera-Marschitz, M. Goiny, H. Utsumi, S. Ferre, L. Håkansson, A. Nordberg, U. Ungerstedt, Effect of unilateral nucleus basalis lesion on cortical and striatal acetylcholine and dopamine release monitored in vivo with microdialysis. Neurosci. Lett. 110, 172–179 (1990). 2325883 10.1016/0304-3940(90)90807-l

[16] R. G. MacKenzie, M. K. Stachowiak, M. J. Zigmond, Dopaminergic inhibition of striatal acetylcholine release after 6-hydroxydopamine. Eur. J. Pharmacol. 168, 43–52 (1989). 2511032 10.1016/0014-2999(89)90631-6

[17] J. W. McKinley, Z. Shi, I. Kawikova, M. Hur, I. J. Bamford, S. P. Sudarsana Devi, A. Vahedipour, M. Darvas, N. S. Bamford, Dopamine deficiency reduces striatal cholinergic interneuron function in models of Parkinson’s disease. Neuron 103, 1056–1072.e6 (2019). 31324539 10.1016/j.neuron.2019.06.013 PMC7102938

[18] G. Sanchez, M. J. Rodriguez, P. Pomata, L. Rela, M. G. Murer, Reduction of an afterhyperpolarization current increases excitability in striatal cholinergic interneurons in rat parkinsonism. J. Neurosci. 31, 6553–6564 (2011). 21525296 10.1523/JNEUROSCI.6345-10.2011 PMC6622669

[19] R. Spehlmann, S. M. Stahl, Dopamine acetylcholine imbalance in Parkinson’s disease. Possible regenerative overgrowth of cholinergic axon terminals. Lancet 307, 724–726 (1976). 10.1016/s0140-6736(76)93095-6 56538

[20] S. M. Hersch, C. A. Gutekunst, H. D. Rees, C. J. Heilman, A. I. Levey, Distribution of m1-m4 muscarinic receptor proteins in the rat striatum: Light and electron microscopic immunocytochemistry using subtype-specific antibodies. J. Neurosci. 14, 3351–3363 (1994). 8182478 10.1523/JNEUROSCI.14-05-03351.1994 PMC6577507

[21] A. I. Levey, C. A. Kitt, W. F. Simonds, D. L. Price, M. R. Brann, Identification and localization of muscarinic acetylcholine receptor proteins in brain with subtype-specific antibodies. J. Neurosci. 11, 3218–3226 (1991). 1941081 10.1523/JNEUROSCI.11-10-03218.1991 PMC6575445

[22] A. A. Mamaligas, C. P. Ford, Spontaneous synaptic activation of muscarinic receptors by striatal cholinergic neuron firing. Neuron 91, 574–586 (2016). 27373830 10.1016/j.neuron.2016.06.021 PMC5234077

[23] M. S. Moehle, P. J. Conn, Roles of the M_4_ acetylcholine receptor in the basal ganglia and the treatment of movement disorders. Mov. Disord. 34, 1089–1099 (2019). 31211471 10.1002/mds.27740 PMC6699902

[24] D. J. Foster, J. M. Wilson, D. H. Remke, M. S. Mahmood, M. J. Uddin, J. Wess, S. Patel, L. J. Marnett, C. M. Niswender, C. K. Jones, Z. Xiang, C. W. Lindsley, J. M. Rook, P. J. Conn, Antipsychotic-like effects of M4 positive allosteric modulators are mediated by CB2 receptor-dependent inhibition of dopamine release. Neuron 91, 1244–1252 (2016). 27618677 10.1016/j.neuron.2016.08.017 PMC5033724

[25] J. Jeon, D. Dencker, G. Wörtwein, D. P. D. Woldbye, Y. Cui, A. A. Davis, A. I. Levey, G. Schütz, T. N. Sager, A. Mørk, C. Li, C.-X. Deng, A. Fink-Jensen, J. Wess, A subpopulation of neuronal M_4_ muscarinic acetylcholine receptors plays a critical role in modulating dopamine-dependent behaviors. J. Neurosci. 30, 2396–2405 (2010). 20147565 10.1523/JNEUROSCI.3843-09.2010 PMC2824442

[26] M. S. Moehle, T. Pancani, N. Byun, S. E. Yohn, G. H. Wilson III, J. W. Dickerson, D. H. Remke, Z. Xiang, C. M. Niswender, J. Wess, C. K. Jones, C. W. Lindsley, J. M. Rook, P. J. Conn, Cholinergic projections to the substantia nigra pars reticulata inhibit dopamine modulation of basal ganglia through the M_4_ muscarinic receptor. Neuron 96, 1358–1372.e4 (2017). 29268098 10.1016/j.neuron.2017.12.008 PMC5753765

[27] A. G. Nair, L. R. V. Castro, M. El Khoury, V. Gorgievski, B. Giros, E. T. Tzavara, J. Hellgren-Kotaleski, P. Vincent, The high efficacy of muscarinic M4 receptor in D1 medium spiny neurons reverses striatal hyperdopaminergia. Neuropharmacology 146, 74–83 (2019). 30468798 10.1016/j.neuropharm.2018.11.029

[28] P. Onali, M. C. Olianas, Muscarinic M4 receptor inhibition of dopamine D1-like receptor signalling in rat nucleus accumbens. Eur. J. Pharmacol. 448, 105–111 (2002). 12144929 10.1016/s0014-2999(02)01910-6

[29] W. Shen, J. L. Plotkin, V. Francardo, W. K. D. Ko, Z. Xie, Q. Li, T. Fieblinger, J. Wess, R. R. Neubig, C. W. Lindsley, P. J. Conn, P. Greengard, E. Bezard, M. A. Cenci, D. J. Surmeier, M4 muscarinic receptor signaling ameliorates striatal plasticity deficits in models of L-DOPA-induced dyskinesia. Neuron 88, 762–773 (2015). 26590347 10.1016/j.neuron.2015.10.039 PMC4864040

[30] G. C. Cotzias, P. S. Papavasiliou, R. Gellene, Modification of Parkinsonism—Chronic treatment with L-Dopa. N. Engl. J. Med. 280, 337–345 (1969). 4178641 10.1056/NEJM196902132800701

[31] Y. Cai, C. P. Ford, Dopamine cells differentially regulate striatal cholinergic transmission across regions through corelease of dopamine and glutamate. Cell Rep. 25, 3148–3157.e3 (2018). 30540946 10.1016/j.celrep.2018.11.053 PMC6658127

[32] M. I. Ekstrand, M. Terzioglu, D. Galter, S. Zhu, C. Hofstetter, E. Lindqvist, S. Thams, A. Bergstrand, F. S. Hansson, A. Trifunovic, B. Hoffer, S. Cullheim, A. H. Mohammed, L. Olson, N.-G. Larsson, Progressive Parkinsonism in mice with respiratory-chain-deficient dopamine neurons. Proc. Natl. Acad. Sci. U.S.A. 104, 1325–1330 (2007). 17227870 10.1073/pnas.0605208103 PMC1783140

[33] A. R. Howe, D. J. Surmeier, Muscarinic receptors modulate N-, P-, and L-type Ca2+ currents in rat striatal neurons through parallel pathways. J. Neurosci. 15, 458–469 (1995). 7823150 10.1523/JNEUROSCI.15-01-00458.1995 PMC6578312

[34] M. Day, D. Wokosin, J. L. Plotkin, X. Tian, D. J. Surmeier, Differential excitability and modulation of striatal medium spiny neuron dendrites. J. Neurosci. 28, 11603–11614 (2008). 18987196 10.1523/JNEUROSCI.1840-08.2008 PMC3235729

[35] T. Hernández-Flores, O. Hernández-González, M. B. Pérez-Ramírez, E. Lara-González, M. A. Arias-García, M. Duhne, A. Pérez-Burgos, G. A. Prieto, A. Figueroa, E. Galarraga, J. Bargas, Modulation of direct pathway striatal projection neurons by muscarinic M4-type receptors. Neuropharmacology 89, 232–244 (2015). 25290553 10.1016/j.neuropharm.2014.09.028

[36] V. Avilés-Rosas, E. Rendón-Ochoa, T. Hernández-Flores, M. Flores-León, C. Arias, E. Galarraga, J. Bargas, Role of M4 receptor cholinergic signaling in direct pathway striatal projection neurons during dopamine depletion. Synapse 78, e22287 (2024). 38427384 10.1002/syn.22287

[37] H. Xiao, M. Li, J. Cai, N. Li, M. Zhou, P. Wen, Z. Xie, Q. Wang, J. Chang, W. Zhang, Selective cholinergic depletion of pedunculopontine tegmental nucleus aggravates freezing of gait in parkinsonian rats. Neurosci. Lett. 659, 92–98 (2017). 28803956 10.1016/j.neulet.2017.08.016

[38] N. Wenger, A. Vogt, M. Skrobot, E. L. Garulli, B. Kabaoglu, C. Salchow-Hömmen, T. Schauer, D. Kroneberg, M. K. Schuhmann, C. W. Ip, C. Harms, M. Endres, I. U. Isaias, P. Tovote, R. Blum, Rodent models for gait network disorders in Parkinson’s disease - a translational perspective. Exp. Neurol. 352, 114011 (2022). 35176273 10.1016/j.expneurol.2022.114011

[39] E. M. Ross, T. M. Wilkie, GTPase-activating proteins for heterotrimeric G proteins: Regulators of G protein signaling (RGS) and RGS-like proteins. Annu. Rev. Biochem. 69, 795–827 (2000). 10966476 10.1146/annurev.biochem.69.1.795

[40] D. M. Berman, T. M. Wilkie, A. G. Gilman, GAIP and RGS4 are GTPase-activating proteins for the Gi subfamily of G protein alpha subunits. Cell 86, 445–452 (1996). 8756726 10.1016/s0092-8674(00)80117-8

[41] I. Masuho, S. Balaji, B. S. Muntean, N. K. Skamangas, S. Chavali, J. J. G. Tesmer, M. M. Babu, K. A. Martemyanov, A global map of G protein signaling regulation by RGS proteins. Cell 183, 503–521.e19 (2020). 33007266 10.1016/j.cell.2020.08.052 PMC7572916

[42] M. Geurts, J.-M. Maloteaux, E. Hermans, Altered expression of regulators of G-protein signaling (RGS) mRNAs in the striatum of rats undergoing dopamine depletion. Biochem. Pharmacol. 66, 1163–1170 (2003). 14505795 10.1016/s0006-2952(03)00447-7

[43] T. N. Lerner, A. C. Kreitzer, RGS4 is required for dopaminergic control of striatal LTD and susceptibility to Parkinsonian motor deficits. Neuron 73, 347–359 (2012). 22284188 10.1016/j.neuron.2011.11.015 PMC3269032

[44] J.-M. Taymans, H. K. Kia, R. Claes, C. Cruz, J. Leysen, X. Langlois, Dopamine receptor-mediated regulation of RGS2 and RGS4 mRNA differentially depends on ascending dopamine projections and time. Eur. J. Neurosci. 19, 2249–2260 (2004). 15090051 10.1111/j.0953-816X.2004.03336.x

[45] K. Avrampou, K. D. Pryce, A. Ramakrishnan, F. Sakloth, S. Gaspari, R. A. Serafini, V. Mitsi, C. Polizu, C. Swartz, B. Ligas, A. Richards, L. Shen, F. B. Carr, V. Zachariou, RGS4 maintains chronic pain symptoms in rodent models. J. Neurosci. 39, 8291–8304 (2019). 31308097 10.1523/JNEUROSCI.3154-18.2019 PMC6794935

[46] M.-H. Han, W. Renthal, R. H. Ring, Z. Rahman, K. Psifogeorgou, D. Howland, S. Birnbaum, K. Young, R. Neve, E. J. Nestler, V. Zachariou, Brain region specific actions of regulator of G protein signaling 4 oppose morphine reward and dependence but promote analgesia. Biol. Psychiatry 67, 761–769 (2010). 19914603 10.1016/j.biopsych.2009.08.041 PMC3077672

[47] M. Stratinaki, A. Varidaki, V. Mitsi, S. Ghose, J. Magida, C. Dias, S. J. Russo, V. Vialou, B. J. Caldarone, C. A. Tamminga, E. J. Nestler, V. Zachariou, Regulator of G protein signaling 4 is a crucial modulator of antidepressant drug action in depression and neuropathic pain models. Proc. Natl. Acad. Sci. U.S.A. 110, 8254–8259 (2013). 23630294 10.1073/pnas.1214696110 PMC3657820

[48] A. Ashrafi, P. Garcia, H. Kollmus, K. Schughart, A. Del Sol, M. Buttini, E. Glaab, Absence of regulator of G-protein signaling 4 does not protect against dopamine neuron dysfunction and injury in the mouse 6-hydroxydopamine lesion model of Parkinson’s disease. Neurobiol. Aging 58, 30–33 (2017). 28697377 10.1016/j.neurobiolaging.2017.06.008

[49] M. Feyder, A. Bonito Oliva, G. Fisone, L-DOPA-Induced dyskinesia and abnormal signaling in striatal medium spiny neurons: Focus on dopamine D1 receptor-mediated transmission. Front. Behav. Neurosci. 5, 71 (2011). 22028687 10.3389/fnbeh.2011.00071 PMC3199545

[50] A. E. Girasole, M. Y. Lum, D. Nathaniel, C. J. Bair-Marshall, C. J. Guenthner, L. Luo, A. C. Kreitzer, A. B. Nelson, A subpopulation of striatal neurons mediates levodopa-induced dyskinesia. Neuron 97, 787–795.e6 (2018). 29398356 10.1016/j.neuron.2018.01.017 PMC6233726

[51] B. Picconi, D. Centonze, K. Håkansson, G. Bernardi, P. Greengard, G. Fisone, M. A. Cenci, P. Calabresi, Loss of bidirectional striatal synaptic plasticity in L-DOPA–induced dyskinesia. Nat. Neurosci. 6, 501–506 (2003). 12665799 10.1038/nn1040

[52] M. B. Ryan, C. Bair-Marshall, A. B. Nelson, Aberrant striatal activity in parkinsonism and levodopa-induced dyskinesia. Cell Rep. 23, 3438–3446.e5 (2018). 29924988 10.1016/j.celrep.2018.05.059 PMC6407866

[53] G. Spigolon, G. Fisone, Signal transduction in L-DOPA-induced dyskinesia: From receptor sensitization to abnormal gene expression. J. Neural Transm. 125, 1171–1186 (2018). 29396608 10.1007/s00702-018-1847-7 PMC6060907

[54] M. A. Cenci, M. Lundblad, Ratings of L-DOPA-Induced dyskinesia in the unilateral 6-OHDA lesion model of Parkinson’s disease in rats and mice. Curr. Protoc. Neurosci. 41, 9.25.1–9.25.23 (2007). 10.1002/0471142301.ns0925s41 18428668

[55] S. H. Fox, R. Katzenschlager, S.-Y. Lim, B. Barton, R. M. A. De Bie, K. Seppi, M. Coelho, C. Sampaio, Movement Disorder Society Evidence-Based Medicine Committee, International Parkinson and movement disorder society evidence-based medicine review: Update on treatments for the motor symptoms of Parkinson’s disease. Mov. Disord. 33, 1248–1266 (2018). 29570866 10.1002/mds.27372

[56] R. Katzenschlager, C. Sampaio, J. Costa, A. Lees, Anticholinergics for symptomatic management of Parkinson’s disease. Cochrane Database Syst. Rev. 2003, CD003735 (2002). 10.1002/14651858.CD003735 PMC8728160 12804486

[57] N. Kayadjanian, W. N. Schofield, J. Andren, D. J. Sirinathsinghji, M.-J. Besson, Cortical and nigral deafferentation and striatal cholinergic markers in the rat dorsal striatum: Different effects on the expression of mRNAs encoding choline acetyltransferase and muscarinic m1 and m4 receptors. Eur. J. Neurosci. 11, 3659–3668 (1999). 10564373 10.1046/j.1460-9568.1999.00788.x

[58] L. Shan, O. Diaz, Y. Zhang, B. Ladenheim, J.-L. Cadet, Y.-H. Chiang, L. Olson, B. J. Hoffer, C. M. Bäckman, L-Dopa induced dyskinesias in Parkinsonian mice: Disease severity or L-Dopa history. Brain Res. 1618, 261–269 (2015). 26086365 10.1016/j.brainres.2015.06.005 PMC4710145

[59] T. Mann, K. Zilles, F. Klawitter, M. Cremer, A. Hawlitschka, N. Palomero-Gallagher, O. Schmitt, A. Wree, Acetylcholine neurotransmitter receptor densities in the striatum of hemiparkinsonian rats following botulinum neurotoxin-A injection. Front. Neuroanat. 12, 65 (2018). 30147647 10.3389/fnana.2018.00065 PMC6095974

[60] V. Bernard, A. I. Levey, B. Bloch, Regulation of the subcellular distribution of m4 muscarinic acetylcholine receptors in striatal neurons in vivo by the cholinergic environment: Evidence for regulation of cell surface receptors by endogenous and exogenous stimulation. J. Neurosci. 19, 10237–10249 (1999). 10575021 10.1523/JNEUROSCI.19-23-10237.1999 PMC6782421

[61] I. Liste, V. Bernard, B. Bloch, Acute and chronic acetylcholinesterase inhibition regulates in vivo the localization and abundance of muscarinic receptors m2 and m4 at the cell surface and in the cytoplasm of striatal neurons. Mol. Cell. Neurosci. 20, 244–256 (2002). 12093157 10.1006/mcne.2001.1083

[62] B. A. Habecker, N. M. Nathanson, Regulation of muscarinic acetylcholine receptor mRNA expression by activation of homologous and heterologous receptors. Proc. Natl. Acad. Sci. U.S.A. 89, 5035–5038 (1992). 1594610 10.1073/pnas.89.11.5035 PMC49223

[63] M. Geurts, E. Hermans, J.-M. Maloteaux, Opposite modulation of regulators of G protein signalling-2 (RGS2) and RGS4 expression by dopamine receptors in the rat striatum. Neurosci. Lett. 333, 146–150 (2002). 12419501 10.1016/s0304-3940(02)01004-2

[64] D. J. Pepperl, S. Shah-Basu, D. VanLeeuwen, J. G. Granneman, R. G. MacKenzie, Regulation of RGS mRNAs by cAMP in PC12 Cells. Biochem. Biophys. Res. Commun. 243, 52–55 (1998). 9473478 10.1006/bbrc.1997.8056

[65] J.-M. Taymans, J. E. Leysen, X. Langlois, Striatal gene expression of RGS2 and RGS4 is specifically mediated by dopamine D1 and D2 receptors: Clues for RGS2 and RGS4 functions. J. Neurochem. 84, 1118–1127 (2003). 12603835 10.1046/j.1471-4159.2003.01610.x

[66] J. L. McKay, F. C. Goldstein, B. Sommerfeld, D. Bernhard, S. Perez Parra, S. A. Factor, Freezing of Gait can persist after an acute levodopa challenge in Parkinson’s disease. NPJ Parkinsons Dis. 5, 25 (2019). 31799377 10.1038/s41531-019-0099-z PMC6874572

[67] K. Sethi, Levodopa unresponsive symptoms in Parkinson disease. Mov. Disord. 23, S521–S533 (2008). 18781679 10.1002/mds.22049

[68] N. I. Bohnen, A. J. Yarnall, R. S. Weil, E. Moro, M. S. Moehle, P. Borghammer, M.-A. Bedard, R. L. Albin, Cholinergic system changes in Parkinson’s disease: Emerging therapeutic approaches. Lancet Neurol. 21, 381–392 (2022). 35131038 10.1016/S1474-4422(21)00377-X PMC8985079

[69] N. I. Bohnen, P. Kanel, R. A. Koeppe, C. A. Sanchez-Catasus, K. A. Frey, P. Scott, G. M. Constantine, R. L. Albin, M. L. T. M. Müller, Regional cerebral cholinergic nerve terminal integrity and cardinal motor features in Parkinson’s disease. Brain Commun. 3, fcab109 (2021). 34704022 10.1093/braincomms/fcab109 PMC8196256

[70] J. Pasquini, D. J. Brooks, N. Pavese, The cholinergic brain in Parkinson’s disease. Mov. Disord. Clin. Pract. 8, 1012–1026 (2021). 34631936 10.1002/mdc3.13319 PMC8485627

[71] C. Liu, X. Cai, A. Ritzau-Jost, P. F. Kramer, Y. Li, Z. M. Khaliq, S. Hallermann, P. S. Kaeser, An action potential initiation mechanism in distal axons for the control of dopamine release. Science 375, 1378–1385 (2022). 35324301 10.1126/science.abn0532 PMC9081985

[72] M. Howe, I. Ridouh, A. L. Allegra Mascaro, A. Larios, M. Azcorra, D. A. Dombeck, Coordination of rapid cholinergic and dopaminergic signaling in striatum during spontaneous movement. eLife 8, e44903 (2019). 30920369 10.7554/eLife.44903 PMC6457892

[73] C. Avila, A. Kucinski, M. Sarter, Complex movement control in a rat model of parkinsonian falls: Bidirectional control by striatal cholinergic interneurons. J. Neurosci. 40, 6049–6067 (2020). 32554512 10.1523/JNEUROSCI.0220-20.2020 PMC7392507

[74] M. S. Moehle, A. M. Bender, J. W. Dickerson, D. J. Foster, A. Qi, H. P. Cho, Y. Donsante, W. Peng, Z. Bryant, K. J. Stillwell, T. M. Bridges, S. Chang, K. J. Watson, J. C. O’Neill, J. L. Engers, L. Peng, A. L. Rodriguez, C. M. Niswender, C. W. Lindsley, E. J. Hess, P. J. Conn, J. M. Rook, Discovery of the first selective M4 muscarinic acetylcholine receptor antagonists with in vivo antiparkinsonian and antidystonic efficacy. ACS Pharmacol. Transl. Sci. 4, 1306–1321 (2021). 34423268 10.1021/acsptsci.0c00162 PMC8369681

[75] R. M. Paz, M. G. Murer, Mechanisms of antiparkinsonian anticholinergic therapy revisited. Neuroscience 467, 201–217 (2021). 34048797 10.1016/j.neuroscience.2021.05.026

[76] A. Brugnoli, C. A. Pisanò, M. Morari, Striatal and nigral muscarinic type 1 and type 4 receptors modulate levodopa-induced dyskinesia and striato-nigral pathway activation in 6-hydroxydopamine hemilesioned rats. Neurobiol. Dis. 144, 105044 (2020). 32798726 10.1016/j.nbd.2020.105044

[77] L. L. Blazer, A. J. Storaska, E. M. Jutkiewicz, E. M. Turner, M. Calcagno, S. M. Wade, Q. Wang, X.-P. Huang, J. R. Traynor, S. M. Husbands, M. Morari, R. R. Neubig, Selectivity and anti-Parkinson’s potential of thiadiazolidinone RGS4 inhibitors. ACS Chem. Nerosci. 6, 911–919 (2015). 10.1021/acschemneuro.5b00063 25844489

[78] W. K. D. Ko, M.-L. Martin-Negrier, E. Bezard, A. R. Crossman, P. Ravenscroft, RGS4 is involved in the generation of abnormal involuntary movements in the unilateral 6-OHDA-lesioned rat model of Parkinson’s disease. Neurobiol. Dis. 70, 138–148 (2014). 24969021 10.1016/j.nbd.2014.06.013

[79] C. A. Sanchez-Catasus, N. I. Bohnen, N. D’Cruz, M. L. T. M. Müller, Striatal acetylcholine-dopamine imbalance in Parkinson disease: In vivo neuroimaging study with dual-tracer PET and dopaminergic PET-informed correlational tractography. J. Nucl. Med. 63, 438–445 (2022). 34272323 10.2967/jnumed.121.261939 PMC8978203

[80] A. C. Krok, M. Maltese, P. Mistry, X. Miao, Y. Li, N. X. Tritsch, Intrinsic dopamine and acetylcholine dynamics in the striatum of mice. Nature 621, 543–549 (2023). 37558873 10.1038/s41586-023-05995-9 PMC11577287

[81] R. T. Pressler, B. W. Strowbridge, Functional specialization of interneuron dendrites: Identification of action potential initiation zone in axonless olfactory bulb granule cells. J. Neurosci. 39, 9674–9688 (2019). 31662426 10.1523/JNEUROSCI.1763-19.2019 PMC6891067

