# Supplementary material for: Reduced striatal M4-cholinergic signaling following dopamine loss contributes to parkinsonian and l-DOPA–induced dyskinetic behaviors
Source: Sci Adv. 2024 Nov 20;10(47):eadp6301. doi: 10.1126/sciadv.adp6301 (PMC11578179; doi:10.1126/sciadv.adp6301)
Supplement: Supplementary file 1 — Figs. S1 to S5 Table S1 [file sciadv.adp6301_sm.pdf]

Science Advances
AAAS

Supplementary Materials for

Reduced striatal M4-cholinergic signaling following dopamine loss contributes to parkinsonian and L-DOPA–induced dyskinetic behaviors

Beatriz E. Nielsen and Christopher P. Ford

Corresponding author: Christopher P. Ford, christopher.ford@cuanschutz.edu

Sci. Adv. **10**, eadp6301 (2024)
DOI: 10.1126/sciadv.adp6301

This PDF file includes:

Figs. S1 to S5
Table S1

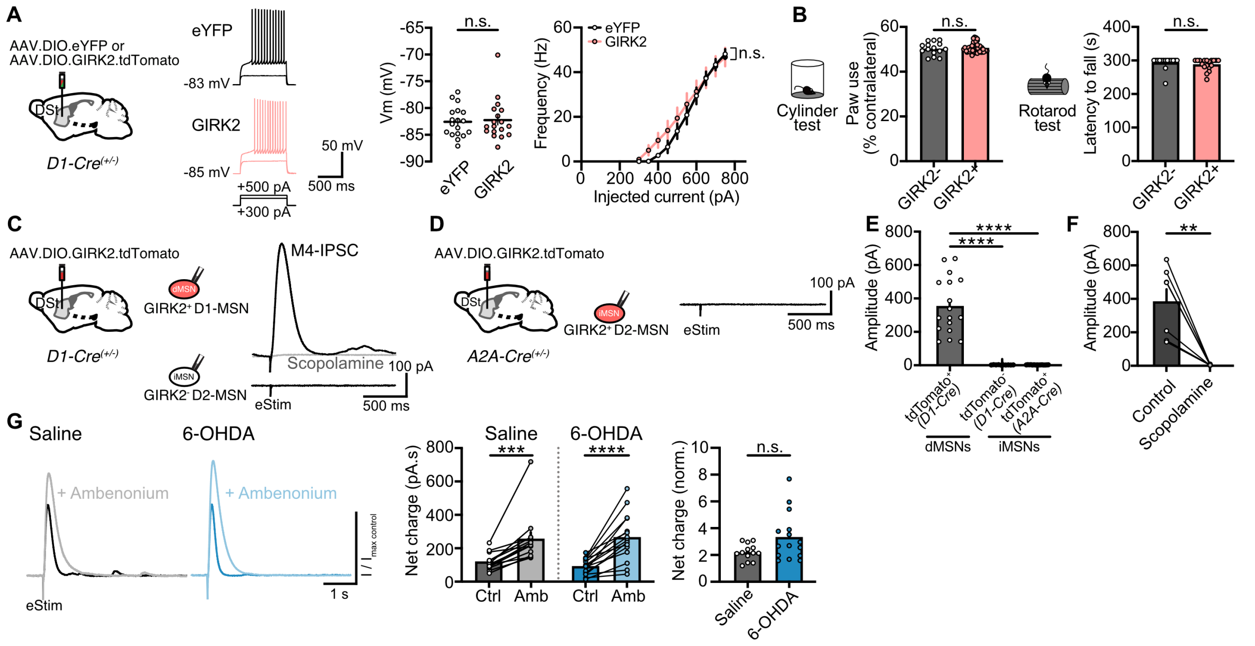

Fig. S1 (Related to Fig. 1). Extended characterization of GIRK2 overexpression and the effects of blocking acetylcholinesterase.

- **(A)** Schematic of AAV9.hSyn.DIO.tdTomato.T2A.GIRK2 or AAV.DIO.eYFP injections into the DSt of D1-Cre mice (left). Current-clamp representative recordings of dMSNs expressing eYFP or TdTomato and GIRK2 (center). Quantification of resting membrane potential (n=18, N=3 per condition; p=0.9938; unpaired t-test) and excitability (right) (n=15, N=3 per condition; p=0.4596 for group effect; Two-Way RM ANOVA).
- **(B)** Cylinder and rotarod test performances for mice unilaterally expressing or non-expressing GIRK2 (cylinder test: GIRK2$^-$ N=15, GIRK2$^+$ N=49; p=0.9219; Mann-Whitney / rotarod test: GIRK2$^-$ N=15, GIRK2$^+$ N=18; p=0.0603; Mann-Whitney).
- **(C)** Schematic of AAV9.hSyn.DIO.tdTomato.T2A.GIRK2 injection into the DSt of D1-Cre mice (left) and representative trace of electrically evoked M4-IPSC recorded from tdTomato$^+$ dMSNs (black), blocked by muscarinic antagonist scopolamine (1 µM) (gray) (top right). No M4-IPSCs are detected in tdTomato$^-$ putative ‘iMSNs’ (bottom right).
- **(D)** Schematic of AAV9.hSyn.DIO.tdTomato.T2A.GIRK2 injection into the DSt of A2A-Cre mice (left) and representative recording of tdTomato$^+$ iMSNs showing the absence of M4-IPSCs after electrical stimulation (right).
- **(E)** Summary data comparing tdTomato$^+$ MSNs from D1-Cre mice (n=17, N=6), tdTomato$^-$ MSNs from D1-Cre mice (n=10, N=3) and tdTomato$^+$ MSNs from A2A-Cre mice (n=10, N=3) (p<0.0001; Kruskal-Wallis, Dunn’s post-hoc test).
- **(F)** Quantification of scopolamine effect in D (n=7, N=3; p=0.0029; paired t-test).
- **(G)** Representative traces of electrically evoked M4-IPSCs before (Ctrl) and after (Amb) bath application of ambenonium (10 nM) (left). Quantification of net charge, absolute values (center) and normalized to control (right) (saline: n=13, N=7; p=0.0002; Wilcoxon; 6-OHDA: n=15, N=8; p<0.0001; paired t-test / Normalized data: p=0.0799; Mann-Whitney).

Summary data is mean ± SEM. Extended statistical data is provided in Supplemental Table S1. n: number of cells, N: number of mice; n.s. p>0.05; *p<0.05; **p<0.01; ***p<0.001; ****p<0.0001.

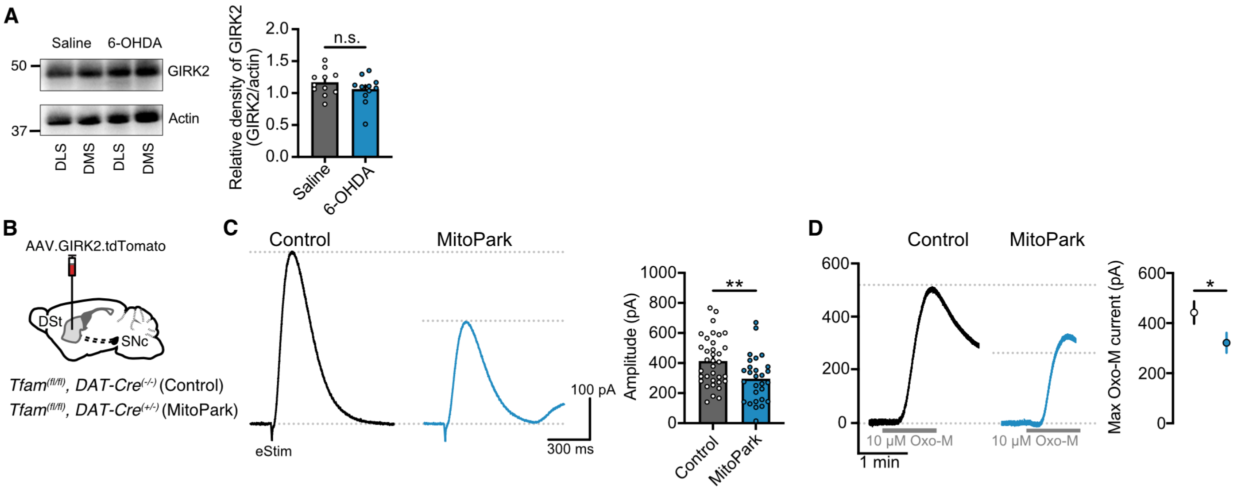

Fig. S2 (Related to Fig. 2). Quantification of GIRK2 expression levels and M4-signaling in MitoPark mice.

- **(A)** Representative western blot for GIRK2 levels and quantification normalized to actin (N=11 for each condition; p=0.2575; unpaired t-test).
- **(B)** Schematics of AAV9.hSyn.tdTomato.T2A injections into the DSt of MitoPark (*Tfam*$^{(fl/fl)}$, *DAT-Cre*$^{(+/-)}$) and littermate controls (*(Tfam*$^{(fl/fl)}$*, DAT-Cre*$^{(-/-)}$*)*
- **(C)** Representative traces and quantification of electrically evoked M4-IPSCs (Control: n=35, N=6; MitoPark: n=28, N=5; p=0.0051; unpaired t-test).
- **(D)** Representative traces of M4-mediated Oxo-M currents following bath application of Oxo-M (10 µM). Spontaneous M4-IPSCs and electrical artifacts were blanked for clarity (left). Maximal Oxo-M current summary values (right) (control: n=23, N=6; 6-OHDA: n=22, N=5; p=0.0202; unpaired t-test).

Summary data is mean ± SEM. Extended statistical data is provided in Supplemental Table S1. n: number of cells, N: number of mice; n.s. p>0.05; *p<0.05; **p<0.01; ***p<0.001; ****p<0.0001.

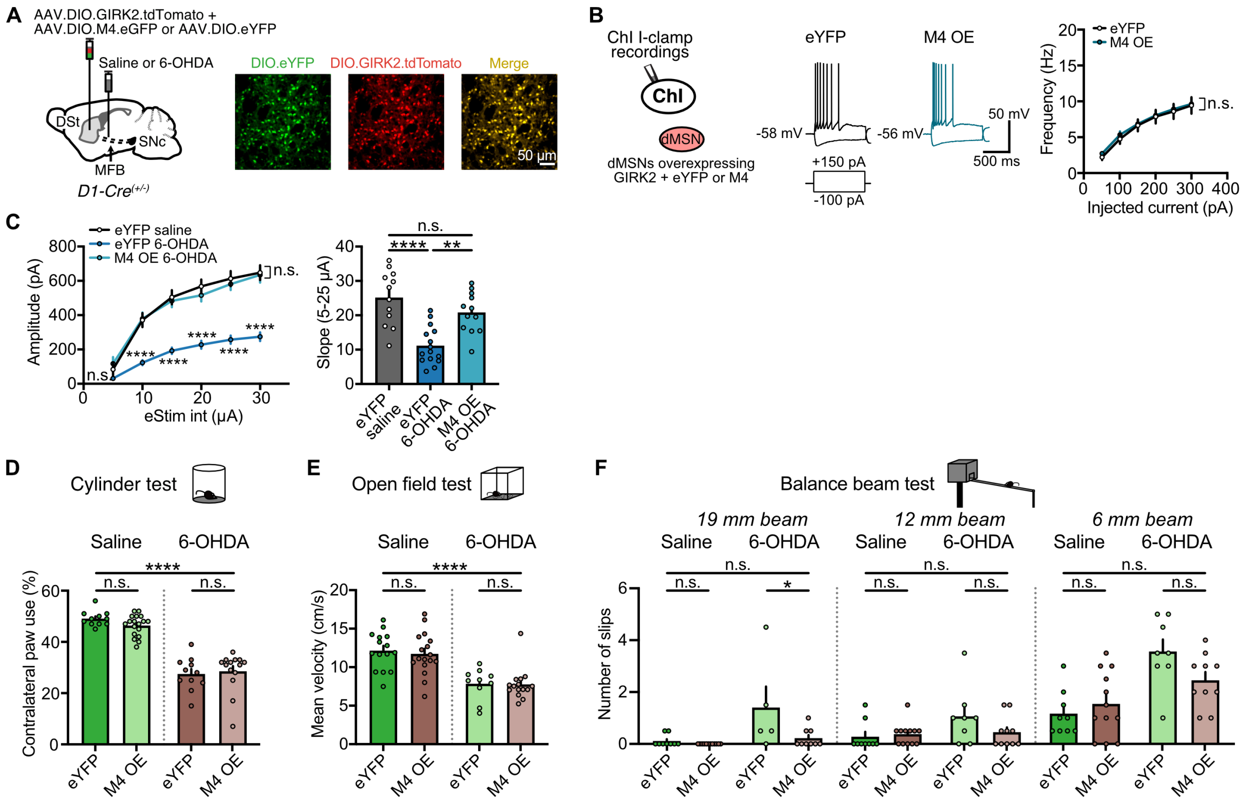

Fig. S3 (Related to Fig. 3). Extended quantification of the effects of M4-receptor overexpression.

(A) Schematics of AAV.DIO.M4.eGFP or AAV.DIO.eYFP co-injections with AAV9.hSyn.DIO.tdTomato.T2A.GIRK2 into the DSt, and saline/6-OHDA injections into the MFB of D1-Cre mice (left). Close-up images of dMSNs co-expressing eYFP and tdTomato fluorescent reporters (right).

(B) Current-clamp representative recordings of ChIs from mice co-expressing GIRK2 with eYFP or M4-receptor selectively in dMSNs (left). Quantification of excitability (right) (eYFP saline: n=21, N=3; M4 OE saline: n=22, N=3; p=0.7365 for group effect; Two-Way RM ANOVA).

(C) Plot of M4-IPSCs amplitudes versus electrical stimulation intensity (eYFP saline: n=12, N=6; eYFP 6-OHDA: n=15, N=5; M4 OE 6-OHDA: n=12-13, N=7; p<0.0001 for group effect; Mixed-model ANOVA, Holm-Šídák's post-hoc test) and summary data of slope for 5-25 µA range (p<0.0001; One-Way ANOVA, Tukey's post-hoc test).

(D) Summary data of cylinder test performance (eYFP saline: N=11; M4 OE saline: N=18; eYFP 6-OHDA: N=11; M4 OE 6-OHDA: N=14; p<0.0001 for treatment and p=0.5968 for group effects; Two-Way ANOVA, Šídák's post-hoc test).

(E) Quantification of mean velocity in open field test (eYFP saline: N=15; M4 OE saline: N=17; eYFP 6-OHDA: N=10; M4 OE 6-OHDA: N=15; p<0.0001 for treatment and p=0.9574 for group effects; Two-Way ANOVA, Šídák's post-hoc test).

(F) Average number of foot slips in the balance beam test for all beams (eYFP saline: N=9; M4 OE saline: N=12; eYFP 6-OHDA: N=5-8; M4 OE 6-OHDA: N=9-10; Two-Way ANOVA, Šídák's post-hoc test for all cases) (19 mm-beam: p=0.0042 for treatment and p=0.0129 for group effects / 12 mm-beam: p=0.0618 for treatment and p=0.2553 for group effects / 6 mm-beam: p<0.0001 for treatment and p=0.3204 for group effects).

Summary data is mean ± SEM. Extended statistical data is provided in Supplemental Table S1. n: number of cells, N: number of mice; n.s. $p>0.05$; *$p<0.05$; **$p<0.01$; ***$p<0.001$; ****$p<0.0001$.

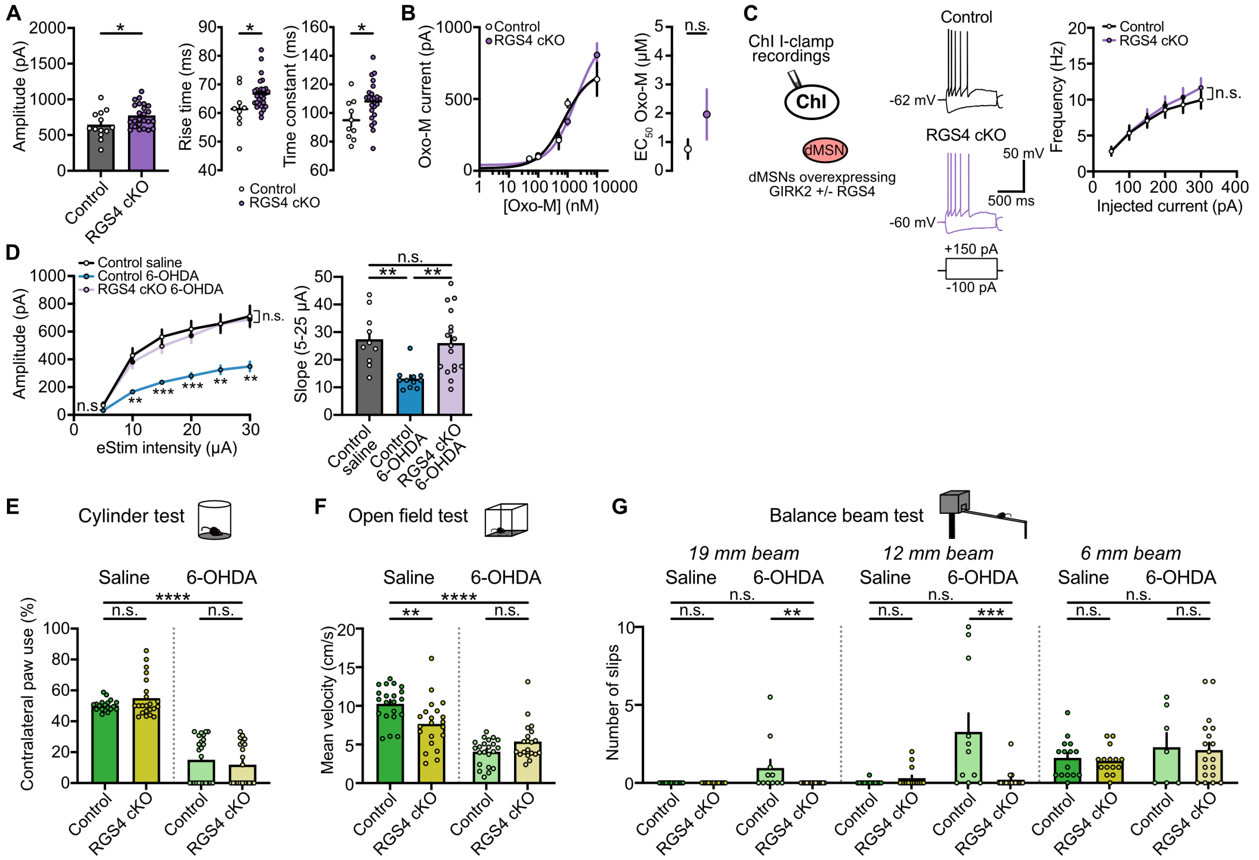

Fig. S4 (Related to Fig. 4). Extended quantification of the effects of selective loss of RGS4 in dMSNs.

(A) Quantification of electrically evoked M4-IPSC amplitudes (25 µA, 0.5 ms), rise time (10-90%) and time constant (15 µA, 0.5 ms) for control and RGS4 cKO saline conditions (Amplitudes (data taken from Figure 4C): control saline: n=13, N=7; RGS4 cKO saline: n=24, N=11; p=0.0369; unpaired t-test / Rise time: control saline: n=9, N=5; RGS4 cKO saline: n=28, N=11; p=0.0304; Mann-Whitney / Time constant: control saline: n=10, N=6; RGS4 cKO saline: n=23, N=11; p=0.0191; unpaired t-test).

(B) Oxo-M concentration-response curve for M4-receptor in control and RGS4 cKO saline conditions (left) with EC_{50} values (right) (control saline: n=22, N=4-5; RGS4 cKO saline: n=29, N=4-10; p=0.2552; unpaired t-test).

(C) Current-clamp representative recordings of ChIs from control and RGS4 cKO mice expressing GIRK2 selectively in dMSNs (left). Quantification of excitability (right) (control saline: n=22, N=3; RGS4 cKO saline: n=21, N=4; p=0.6649 for group effect; Two-Way RM ANOVA).

(D) Plot of M4-IPSC amplitudes versus electrical stimulation intensity (control saline: n=10, N=6; control 6-OHDA: n=11-12, N=6; RGS4 cKO 6-OHDA: n=17-19, N=8; p=0.0005 for group effect; Mixed-model ANOVA, Holm-Šídák's post-hoc test) and summary data of slope for 5-25 µA range (p=0.0006; Kruskal-Wallis, Dunn's post-hoc test).

(E) Summary data of cylinder test performance (control saline: N=19; RGS4 cKO saline: N=22; control 6-OHDA: N=24; RGS4 cKO 6-OHDA: N=21; p<0.0001 for treatment and p=0.8403 for group effects; Two-Way ANOVA, Šídák's post-hoc test).

(F) Quantification of mean velocity in open field test (control saline: N=21; RGS4 cKO saline: N=21; control 6-OHDA: N=22; RGS4 cKO 6-OHDA: N=20; $p<0.0001$ for treatment and $p=0.2292$ for group effects; Two-Way ANOVA, Šídák's post-hoc test).

(G) Average number of foot slips in the balance beam test for all beams (control saline: N=15; RGS4 cKO saline: N=15; control 6-OHDA: N=7-11; RGS4 cKO 6-OHDA: N=17-19; Two-Way ANOVA, Šídák's post-hoc test for all cases) (19 mm-beam: $p=0.021$ for treatment and group effects / 12 mm-beam: $p=0.0008$ for treatment and $p=0.0015$ for group effects / 6 mm-beam: $p=0.1459$ for treatment and $p=0.6881$ for group effects).

Summary data is mean ± SEM. Extended statistical data is provided in Supplemental Table S1. n: number of cells, N: number of mice; n.s. $p>0.05$; *$p<0.05$; **$p<0.01$; ***$p<0.001$; ****$p<0.0001$.

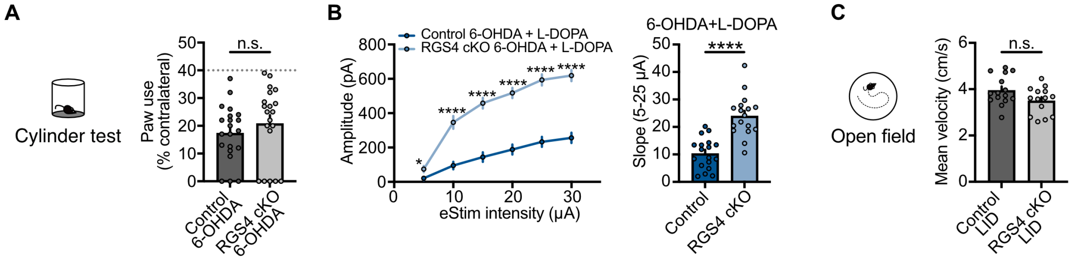

Fig. S5 (Related to Fig. 5). Extended quantification of the effects of L-DOPA on M4-receptor signaling and motor behavior in parkinsonian mice lacking RGS4 in dMSNs.

- **(A)** Summary data of cylinder test performance for control and RGS4 cKO 6-OHDA groups to assess DA depletion before L-DOPA administration (control 6-OHDA: N=21; RGS4 cKO 6-OHDA: N=20; p=0.1528; Mann-Whitney).
- **(B)** Plot of M4-IPSC amplitudes versus electrical stimulation intensity (control 6-OHDA+L-DOPA: n=18, N=5; RGS4 cKO 6-OHDA+L-DOPA: n=17-18, N=4; p<0.0001 for group effect; Mixed-model ANOVA, Holm-Šídák's post-hoc test) and summary data of slope for 5-25 µA range (p<0.0001; unpaired t-test).
- **(C)** Quantification of mean velocity in the open field (control LID: N=15; RGS4 cKO LID: N=14; p=0.0575; unpaired t-test).

Summary data is mean ± SEM. Extended statistical data is provided in Supplemental Table S1. n: number of cells, N: number of mice; n.s. p>0.05; *p<0.05; **p<0.01; ***p<0.001; ****p<0.0001.

Table S1. - Extended Statistical Data

Table S1 - Extended Statistical Data

Figure	Section	Variables and units	Conditions	Mean	SEM	n	N	p-value	(*)	Data comparisons	Statistical test	Post-hoc test	Post-hoc comparisons	p-value	(*)
**TABLE S1 - MAIN FIGURES**															
**1**	**B**	DSt TH-fluorescence ratio vs contralateral hemisphere	Saline	1.02	0.04		4	p<0.0001	****		Unpaired t-test				
			6-OHDA	0.15	0.05		5								
		SNc TH-fluorescence ratio vs contralateral hemisphere	Saline	1.03	0.04		4	p<0.0001	****		Unpaired t-test				
			6-OHDA	0.12	0.02		5								
	**C**	Cylinder test Paw use (% contralateral)	Saline	50.49	0.26		60	p<0.0001	****		Mann-Whitney test				
			6-OHDA	27.87	1.54		63								
		Rotarod test Latency to fall (s)	Saline	261.30	7.70		42	p<0.0001	****		Mann-Whitney test				
			6-OHDA	182.30	8.64		57								
	**E**	M4-IPSC amplitude (pA)	control	337.90	46.95	8	3	p<0.0001	****		RM One-Way ANOVA	Holm-Šídák	control vs VU-0255035	p=0.7595	n.s.
			VU-0255035	325.30	51.37	8	3						control vs tropicamide	p<0.001	****
			tropicamide	5.80	0.62	8	3						VU-0255035 vs tropicamide	p<0.001	****
	**F**	M4-IPSC amplitude (pA) eStim intensity: 25μA	Saline	539.60	44.96	43	16	p=0.0045	**		Mann-Whitney test				
			6-OHDA HD	359.40	30.50	39	13								
	**G**	M4-IPSC amplitude (pA)						p<0.0001	****	Effect of eStim intensity	Mixed-model ANOVA with Geisser Greenhouse's correction	Šídák			
								p=0.0002	***	Effect of Treatment					
								p<0.0001	****	Interaction					
		30 μA	Saline	695.70	66.73	23	12							p=0.0016	**
			6-OHDA	392.80	32.19	35	12								
		25 μA	Saline	658.40	64.78	23	12							p=0.0028	**
			6-OHDA	377.80	31.66	35	12								
		20 μA	Saline	614.00	60.42	23	12							p=0.0027	**
			6-OHDA	350.50	30.81	35	12								
		15 μA	Saline	536.10	54.69	23	12							p=0.0076	**
			6-OHDA	318.00	29.67	35	12								
		10 μA	Saline	383.10	46.80	23	12							p=0.0513	n.s.
			6-OHDA	238.00	22.56	35	12								
		5 μA	Saline	93.62	17.60	23	12							p=0.9957	n.s.
			6-OHDA	82.17	12.25	35	12								
		Slope (5-25 μA)	Saline	27.21	2.77	23	12	p<0.0001	****		Mann-Whitney test				
			6-OHDA	13.67	1.25	35	12								
**2**	**B**	EC50 Oxo-M (μM)	Saline	0.81	0.34	54	5-12	p=0.8348	n.s.		Unpaired t-test				
			6-OHDA	0.72	0.30	42	5-9								
		Max Oxo-M current (pA)	Saline	574.60	90.31	12	10	p<0.0001	****		Mann-Whitney test				
			6-OHDA	251.80	19.58	11	9								
	**C**	M4 fluorescence ratio vs contralateral hemisphere	Saline	0.98	0.02		4	p=0.0139	*		Unpaired t-test				
			6-OHDA	0.85	0.03		6								
	**F**	bAP-evoked Ca2+-transients (ΔF/F)	Saline baseline	1.00	0.14	10	5	p<0.0001	****	Saline baseline vs Saline Oxo-M	Paired t-test				
			Saline Oxo-M	0.82	0.12	10	5								
			6-OHDA baseline	1.00	0.11	9	4	p=0.6206	n.s.	6-OHDA baseline vs 6-OHDA Oxo-M	Paired t-test				
			6-OHDA Oxo-M	1.01	0.13	9	4								
		M4-mediated VGCC modulation (%)	Saline	-19.01	1.62	10	5	p<0.0001	****		Unpaired t-test				
			6-OHDA	1.02	2.78	9	4								
**3**	**A**	M4 fluorescence ratio vs contralateral hemisphere	Saline	0.98	0.02		4	p=0.0017	**		One-way ANOVA	Šídák	Saline vs M4OE saline	p=0.0044	**
			M4 OE saline	1.26	0.04		4						Saline vs M4OE 6OHDA	p=0.0031	**
			M4 OE 6-OHDA	1.25	0.05		6						M4OE saline vs M4OE 6OHDA	p=0.9962	n.s.
	**C**	M4-IPSC amplitude (pA) eStim intensity: 25μA	eYFP saline	592.40	45.53	13	6	p<0.0001	****	Effect of treatment	Two-way ANOVA	Šídák	eYFP saline vs M4 OE saline	p<0.0001	****
			M4 OE saline	733.40	26.54	24	8	p<0.0001	****	Effect of group			eYFP 6-OHDA vs M4 OE 6-OHDA	p<0.0001	****
			eYFP 6-OHDA	268.40	25.80	16	5						eYFP saline vs M4 OE 6-OHDA	p=0.9966	n.s.
			M4 OE 6-OHDA	585.90	30.61	18	7								
	**D**	Max Oxo-M current (pA)	eYFP saline	530.80	44.54	13	6	p<0.0001	****	Effect of treatment	Two-way ANOVA	Šídák	eYFP saline vs M4 OE saline	p<0.0001	****
			M4 OE saline	705.40	35.15	20	10	p<0.0001	****	Effect of group			eYFP 6-OHDA vs M4 OE 6-OHDA	p<0.0001	****
			eYFP 6-OHDA	273.70	33.09	10	5						eYFP saline vs M4 OE 6-OHDA	p=0.997	n.s.
			M4 OE 6-OHDA	552.80	41.57	9	5								
	**E**	Open field Total distance (cm)	eYFP saline	6411.00	399.80		15	p<0.0001	****	Effect of treatment	Two-way ANOVA	Šídák	eYFP saline vs M4 OE saline	p=0.9967	n.s.
			M4 OE saline	6324.00	340.50		17	p=0.9574	n.s.	Effect of group			eYFP 6-OHDA vs M4 OE 6-OHDA	p=0.9938	n.s.
			eYFP 6-OHDA	4041.00	351.60		10	p=0.7656	n.s.	Interaction			eYFP saline vs M4 OE 6-OHDA	p<0.0001	****
			M4 OE 6-OHDA	4166.00	264.20		15								
		Open field Normalized total distance	eYFP 6-OHDA	0.63	0.05		10	p=0.6433	n.s.		Mann-Whitney test				
			M4 OE 6-OHDA	0.66	0.04		15								
	**F**	Rotarod test Latency to fall (s)	eYFP saline	274.60	9.02		10	p=0.0158	*	Effect of treatment	Two-way ANOVA	Šídák	eYFP saline vs M4 OE saline	p=0.0337	*
			M4 OE saline	213.00	17.07		20	p=0.341	n.s.	Effect of group			eYFP 6-OHDA vs M4 OE 6-OHDA	p=0.4585	n.s.
			eYFP 6-OHDA	188.00	13.82		15	p=0.0067	**	Interaction			eYFP saline vs M4 OE 6-OHDA	p=0.0856	n.s.
			M4 OE 6-OHDA	218.30	15.72		14								
		Rotarod test Normalized latency to fall	eYFP 6-OHDA	0.68	0.05		15	p=0.0006	***		Mann-Whitney test				
			M4 OE 6-OHDA	1.00	0.07		14								
	**G**	Balance beam (19 mm) Latency to cross (s)	eYFP saline	4.11	0.40		9	p<0.0001	****	Effect of treatment	Two-way ANOVA	Šídák	eYFP saline vs M4 OE saline	p>0.05	n.s.
			M4 OE saline	4.29	0.37		12	p=0.0055	**	Effect of group			eYFP 6-OHDA vs M4 OE 6-OHDA	p=0.0004	***
			eYFP 6-OHDA	41.95	6.00		11	p=0.0049	**	Interaction			eYFP saline vs M4 OE 6-OHDA	p=0.1777	n.s.
			M4 OE 6-OHDA	16.86	5.60		11								
		Balance beam (12 mm) Latency to cross (s)	eYFP saline	5.00	0.46		9	p<0.0001	****	Effect of treatment	Two-way ANOVA	Šídák	eYFP saline vs M4 OE saline	p=0.9979	n.s.
			M4 OE saline	4.17	0.30		12	p=0.0009	***	Effect of group			eYFP 6-OHDA vs M4 OE 6-OHDA	p<0.0001	****
			eYFP 6-OHDA	41.86	5.06		11	p=0.0017	**	Interaction			eYFP saline vs M4 OE 6-OHDA	p=0.0837	n.s.
			M4 OE 6-OHDA	16.91	4.74		11								
		Balance beam (6 mm) Latency to cross (s)	eYFP saline	8.94	1.03		9	p<0.0001	****	Effect of treatment	Two-way ANOVA	Šídák	eYFP saline vs M4 OE saline	p>0.05	n.s.
			M4 OE saline	8.96	0.86		12	p=0.0113	*	Effect of group			eYFP 6-OHDA vs M4 OE 6-OHDA	p=0.0014	**
			eYFP 6-OHDA	40.00	5.65		11	p=0.0112	*	Interaction			eYFP saline vs M4 OE 6-OHDA	p=0.1795	n.s.
			M4 OE 6-OHDA	19.64	4.65		11								
		Unsuccessful balance beam trials (%)	eYFP saline	0.00	0.00		9	p=0.0021	**	Effect of treatment	Two-way ANOVA	Šídák	eYFP saline vs M4 OE saline	p>0.05	n.s.
			M4 OE saline	0.00	0.00		12	p=0.0599	n.s.	Effect of group			eYFP 6-OHDA vs M4 OE 6-OHDA	p=0.0243	*
			eYFP 6-OHDA	40.91	12.18		11	p=0.0599	n.s.	Interaction			eYFP saline vs M4 OE 6-OHDA	p=0.7384	n.s.
			M4 OE 6-OHDA	10.61	9.07		11								
**4**	**C**	M4-IPSC amplitude (pA) eStim intensity: 25μA	Control saline	640.30	53.82	13	7	p<0.0001	****	Effect of treatment	Two-way ANOVA	Šídák	Control saline vs RGS4 cKO saline	p<0.0001	****
			RGS4 cKO saline	770.00	32.99	24	11	p<0.0001	****	Effect of group			Control 6-OHDA vs RGS4 cKO 6-OHDA	p<0.0001	****
			Control 6-OHDA	324.10	28.14	13	6						Control saline vs RGS4 cKO 6-OHDA	p=0.9998	n.s.
			RGS4 cKO 6-OHDA	606.80	44.57	29	10								
	**D**	Max Oxo-M current (pA)	Control saline	636.90	105.30	6	5	p=0.0028	**	Effect of treatment	Two-way ANOVA	Šídák	Control saline vs RGS4 cKO saline	p=0.0096	**
			RGS4 cKO saline	806.40	66.80	15	10	p=0.0032	**	Effect of group			Control 6-OHDA vs RGS4 cKO 6-OHDA	p=0.0096	**
			Control 6-OHDA	302.70	20.99	8	5						Control saline vs RGS4 cKO 6-OHDA	p>0.05	n.s.
			RGS4 cKO 6-OHDA	622.20	77.44	9	7								
	**E**	Open field Total distance (cm)	Control saline	5698.00	293.70		21	p<0.0001	****	Effect of treatment	Two-way ANOVA	Šídák	Control saline vs RGS4 cKO saline	p=0.0157	*
			RGS4 cKO saline	4456.00	420.00		21	p=0.4591	n.s.	Effect of group			Control 6-OHDA vs RGS4 cKO 6-OHDA	p=0.2045	n.s.
			Control 6-OHDA	2189.00	194.70		22	p=0.0014	**	Interaction			Control saline vs RGS4 cKO 6-OHDA	p<0.0001	****
			RGS4 cKO 6-OHDA	2976.00	280.00		20								
		Open field Normalized total distance	Control 6-OHDA	0.38	0.03		22	p=0.0002	***	Control 6-OHDA vs RGS4 cKO 6OHDA	Mann-Whitney				
			RGS4 cKO 6-OHDA	0.67	0.06		20								
	**F**	Rotarod test Latency to fall (s)	Control saline	230.10	14.69		19	p<0.0001	****	Effect of treatment	Two-way ANOVA	Šídák	Control saline vs RGS4 cKO saline	p=0.0004	***
			RGS4 cKO saline	160.90	10.38		30	p=0.0407	*	Effect of group			Control 6-OHDA vs RGS4 cKO 6-OHDA	p=0.5981	ns
			Control 6-OHDA	111.80	14.53		23	p=0.0004	***	Interaction			Control saline vs RGS4 cKO 6-OHDA	p<0.0001	****
			RGS4 cKO 6-OHDA	130.80	9.26		27								
		Rotarod test Normalized latency to fall (s)	Control 6-OHDA	0.49	0.06		23	p=0.0004	***	Control 6-OHDA vs RGS4 cKO 6OHDA	Unpaired t-test				
			RGS4 cKO 6-OHDA	0.81	0.06		27								
	**G**	Balance beam (19 mm) Latency to cross (s)	Control saline	3.43	0.39		15	p<0.0001	****	Effect of treatment	Two-way ANOVA	Šídák	Control saline vs RGS4 cKO saline	p=0.9988	n.s.
			RGS4 cKO saline	4.17	0.80		15	p=0.024	*	Effect of group			Control 6-OHDA vs RGS4 cKO 6-OHDA	p=0.0007	***
			Control 6-OHDA	47.44	3.53		26	p=0.0142	*	Interaction			Control saline vs RGS4 cKO 6-OHDA	p<0.0001	****
			RGS4 cKO 6-OHDA	30.55	4.30		22								
		Balance beam (12 mm) Latency to cross (s)	Control saline	3.93	0.60		15	p<0.0001	****	Effect of treatment	Two-way ANOVA	Šídák	Control saline vs RGS4 cKO saline	p=0.9584	n.s.
			RGS4 cKO saline	6.27	1.15		15	p=0.0931	n.s.	Effect of group			Control 6-OHDA vs RGS4 cKO 6-OHDA	p=0.0044	**
			Control 6-OHDA	49.10	2.75		26	p=0.0185	*	Interaction			Control saline vs RGS4 cKO 6-OHDA	p<0.0001	****
			RGS4 cKO 6-OHDA	35.52	4.56		22								
		Balance beam (6 mm) Latency to cross (s)	Control saline	10.67	2.00		15	p<0.0001	****	Effect of treatment	Two-way ANOVA	Šídák	Control saline vs RGS4 cKO saline	p=0.9911	n.s.
			RGS4 cKO saline	9.37	2.13		15	p=0.0062	**	Effect of group			Control 6-OHDA vs RGS4 cKO 6-OHDA	p=0.0002	***
			Control 6-OHDA	53.71	2.47		26	p=0.0189	*	Interaction			Control saline vs RGS4 cKO 6-OHDA	p<0.0001	****
			RGS4 cKO 6-OHDA	37.35	3.97		23								
		Unsuccessful balance beam trials (%)	Control saline	0.00	0.00		15	p<0.0001	****	Effect of treatment	Two-way ANOVA	Šídák	Control saline vs RGS4 cKO saline	p>0.05	n.s.
			RGS4 cKO saline	0.00	0.00		15	p=0.0104	*	Effect of group			Control 6-OHDA vs RGS4 cKO 6-OHDA	p=0.0002	***
			Control 6-OHDA	65.38	6.40		26	p=0.0104	*	Interaction			Control saline vs RGS4 cKO 6-OHDA	p=0.0024	**
			RGS4 cKO 6-OHDA	31.88	7.69		23								

Figure	Section	Variables and units	Conditions	Mean	SEM	n	N	p-value	(*)	Data comparisons	Statistical test	Post-hoc test	Post-hoc comparisons	p-value	(*)
5	C	M4-IPSC amplitude (pA)	Control saline	640.30	53.82	13	7	p<0.0001	****		Kruskal-Wallis (ANOVA on ranks)	Dunn	6-OHDA vs 6-OHDA+L-DOPA	p=0.2247	n.s.
			Control 6-OHDA	324.10	28.14	13	6						Saline vs 6-OHDA+L-DOPA	p<0.0001	****
			Control 6-OHDA+L-DOPA	233.20	29.40	18	5								
	D	M4-IPSC amplitude (pA)	RGS4 cKO saline	770.00	32.99	24	11	p<0.0001	****		Kruskal-Wallis (ANOVA on ranks)	Dunn	6-OHDA vs 6-OHDA+L-DOPA	p>0.05	n.s.
			RGS4 cKO 6-OHDA	606.80	44.57	29	10						Saline vs 6-OHDA+L-DOPA	p=0.0088	**
			RGS4 cKO 6-OHDA+L-DOPA	593.70	32.82	18	4								
	E	Open field Total distance (cm)	Control LID	6955.00	296.70		15	p=0.0603	n.s.		Unpaired t-test				
			RGS4 cKO LID	6140.00	289.90		14								
		Contralesional rotations (%)	Control LID	71.65	6.56		15	p=0.5323	n.s.		Mann-Whitney				
			RGS4 cKO LID	57.35	9.21		14								
								p=0.0215	*	Control LID vs 50%	One-sample Wilcoxon Signed Rank Test				
								p=0.4973	n.s.	RGS4 cKO LID vs 50%					
	F	Total AIM score (sum of AOL)						p<0.0001	****	Effect of Time	Two-Way RM ANOVA with Geisser-Greenhouse's correction	Šídák			
								p=0.0001	***	Effect of Group					
								p<0.0001	****	Effect of Subject					
								p=0.0002	***	Interaction					
		0 min	Control LID	0.00	0.00		15								
			RGS4 cKO LID	0.00	0.00		14								
		20 min	Control LID	9.27	0.41		15							p=0.0011	**
			RGS4 cKO LID	6.29	0.53		14								
		40 min	Control LID	8.13	0.70		15							p=0.0118	*
			RGS4 cKO LID	5.00	0.55		14								
		60 min	Control LID	6.33	0.81		15							p=0.0156	*
			RGS4 cKO LID	3.00	0.53		14								
		80 min	Control LID	3.00	0.68		15							p=0.0292	*
			RGS4 cKO LID	0.57	0.31		14								
		100 min	Control LID	1.93	0.84		15							p=0.2295	n.s.
			RGS4 cKO LID	0.00	0.00		14								
		120 min	Control LID	0.40	0.40		15							p=0.9808	n.s.
			RGS4 cKO LID	0.07	0.07		14								
		Peak AIM score (sum of AOL at 20 min)	Control LID	9.27	0.41		15	p=0.0001	***		Unpaired t-test				
			RGS4 cKO LID	6.29	0.53		14								
		Total integrated AIM score (AUC/2h)	Control LID	577.30	53.91		15	p=0.0001	***		Unpaired t-test				
			RGS4 cKO LID	297.90	25.81		14								
**TABLE S1 - SUPPLEMENTAL FIGURES**															
S1	A	Vm (mV)	eYFP	-82.57	2.84	18	3	p=0.9938	n.s.		Unpaired t-test				
			GIRK2	-82.26	3.84	18	3								
		Firing frequency (Hz) dMSN excitability						p<0.0001	****	Effect of injected current	Two-Way RM ANOVA with Geisser-Greenhouse's correction				
								p=0.4596	n.s.	Effect of group					
								p<0.0001	****	Effect of subject					
								p=0.2197	n.s.	Interaction					
		Firing frequency (Hz) Injected current: 300 pA	eYFP	0.00	0.00	15	3								
			GIRK2	1.00	0.68	15	3								
		Firing frequency (Hz) Injected current: 350 pA	eYFP	0.13	0.13	15	3								
			GIRK2	5.03	2.28	15	3								
		Firing frequency (Hz) Injected current: 400 pA	eYFP	2.73	1.50	15	3								
			GIRK2	9.50	3.39	15	3								
		Firing frequency (Hz) Injected current: 450 pA	eYFP	6.53	2.82	15	3								
			GIRK2	13.97	4.34	15	3								
		Firing frequency (Hz) Injected current: 500 pA	eYFP	13.63	3.55	15	3								
			GIRK2	19.40	4.76	15	3								
		Firing frequency (Hz) Injected current: 550 pA	eYFP	20.83	4.18	15	3								
			GIRK2	25.70	4.93	15	3								
		Firing frequency (Hz) Injected current: 600 pA	eYFP	29.37	3.75	15	3								
			GIRK2	31.37	4.89	15	3								
		Firing frequency (Hz) Injected current: 650 pA	eYFP	37.30	3.04	15	3								
			GIRK2	37.53	4.62	15	3								
		Firing frequency (Hz) Injected current: 700 pA	eYFP	43.33	2.57	15	3								
			GIRK2	42.67	4.50	15	3								
		Firing frequency (Hz) Injected current: 750 pA	eYFP	48.10	2.22	15	3								
			GIRK2	46.80	4.15	15	3								
	B	Cylinder test Paw use (% contralateral)	GIRK2-	50.24	0.73		15	p=0.9219	n.s.		Mann-Whitney test				
			GIRK2+	50.55	0.29		49								
		Rotarod test Latency to fall (s)	GIRK2-	293.90	4.74		15	p=0.0603	n.s.		Mann-Whitney test				
			GIRK2+	288.10	3.95		18								
	E	eIPSC amplitude (pA)	tdTomato+ dMSNs D1-Cre	355.90	41.37	17	6	p<0.0001	****		Kruskal-Wallis (ANOVA on ranks)	Dunn			
			tdTomato- 'iMSNs' D1-Cre	2.16	0.24	10.00	3.00						tdTomato+ dMSNs D1-Cre vs tdTomato- 'iMSNs' D1-Cre	p<0.0001	****
			tdTomato- iMSNs A2A-Cre	2.16	0.24	10	3						tdTomato+ dMSNs D1-Cre vs tdTomato+ iMSNs A2A-Cre	p<0.0001	****
	F	eIPSC amplitude (pA)	control	384.60	79.14	7	3	p=0.0029	**		Paired t-test				
			scopolamine	5.33	1.05	7	3								
	G	Net charge (pA.s)	Saline Ctrl	118.80	14.31	13	7	p=0.0002	***	Saline Ctrl vs Saline Amb	Wilcoxon signed rank test				
			Saline Amb	254.90	41.14	13	7								
			6-OHDA Ctrl	90.87	12.53	15	8	p<0.0001	****	6-OHDA Ctrl vs 6-OHDA Amb	Paired t-test				
			6-OHDA Amb	264.50	36.69	15	8								
		Net charge (normalized)	Saline	2.20	0.17	13	7	p=0.0799	n.s.		Mann-Whitney				
			6-OHDA	3.33	0.46	15	8								
S2	A	Relative density of GIRK2 (GIRK2/actin)	Saline DLS	1.16	0.06		11	p=0.2575	n.s.		Unpaired t-test				
			6-OHDA DLS	1.06	0.07		11								
	C	M4-IPSC amplitude (pA) eStim intensity: 25µA	Control	412.70	27.67	35	6	p=0.0051	**		Unpaired t-test				
			MitoPark	295.10	29.19	28	5								
	D	Max Oxo-M current (pA)	Control	448.20	37.52	23	6	p=0.0202	*		Unpaired t-test				
			MitoPark	325.20	34.31	22	5								
S3	B	Firing frequency (Hz) ChI excitability						p<0.0001	****	Effect of injected current	Two-Way RM ANOVA with Geisser-Greenhouse's correction				
								p=0.7365	n.s.	Effect of group					
								p<0.0001	****	Effect of subject					
								p=0.9656	n.s.	Interaction					
		Firing frequency (Hz) Injected current: 50 pA	eYFP saline	2.20	0.41	21	3								
			M4 OE saline	2.71	0.33	22	3								
		Firing frequency (Hz) Injected current: 100 pA	eYFP saline	4.74	0.69	21	3								
			M4 OE saline	5.32	0.47	22	3								
		Firing frequency (Hz) Injected current: 150 pA	eYFP saline	6.64	0.83	21	3								
			M4 OE saline	6.84	0.55	22	3								
		Firing frequency (Hz) Injected current: 200 pA	eYFP saline	7.86	0.96	21	3								
			M4 OE saline	8.04	0.61	22	3								
		Firing frequency (Hz) Injected current: 250 pA	eYFP saline	8.59	1.06	21	3								
			M4 OE saline	8.91	0.65	22	3								
		Firing frequency (Hz) Injected current: 300 pA	eYFP saline	9.42	1.15	21	3								
			M4 OE saline	9.62	0.74	22	3								
	C	M4-IPSCs amplitude (pA)						p<0.0001	****	Effect of eStim intensity	Mixed-model ANOVA with Geisser-Greenhouse's correction	Holm-Šídák			
								p<0.0001	****	Effect of Group					
								p<0.0001	****	Interaction					
		30µA	eYFP saline	647.90	43.42	12	6						eYFP saline vs eYFP 6-OHDA	p<0.0001	****
			eYFP 6-OHDA	274.70	25.62	15	5						eYFP saline vs M4 OE 6-OHDA	p=0.8297	n.s.
			M4 OE 6-OHDA	634.30	44.99	13	7								
		25µA	eYFP saline	613.09	44.08	12	6						eYFP saline vs eYFP 6-OHDA	p<0.0001	****
			eYFP 6-OHDA	256.73	24.58	15	5						eYFP saline vs M4 OE 6-OHDA	p=0.5757	n.s.
			M4 OE 6-OHDA	581.16	34.83	13	7								
		20µA	eYFP saline	567.50	40.35	12	6						eYFP saline vs eYFP 6-OHDA	p<0.0001	****
			eYFP 6-OHDA	228.00	23.33	15	5						eYFP saline vs M4 OE 6-OHDA	p=0.3478	n.s.
			M4 OE 6-OHDA	515.70	35.81	12	7								
		15µA	eYFP saline	504.30	41.25	12	6						eYFP saline vs eYFP 6-OHDA	p<0.0001	****
			eYFP 6-OHDA	192.10	19.13	15	5						eYFP saline vs M4 OE 6-OHDA	p=0.7066	n.s.
			M4 OE 6-OHDA	483.20	36.77	12	7								
		10µA	eYFP saline	371.30	41.09	12	6						eYFP saline vs eYFP 6-OHDA	p=0.0001	****
			eYFP 6-OHDA	123.10	14.54	15	5						eYFP saline vs M4 OE 6-OHDA	p=0.8874	n.s.
			M4 OE 6-OHDA	379.20	36.97	12	7								
		5µA	eYFP saline	83.38	36.04	12	6						eYFP saline vs eYFP 6-OHDA	p=0.3237	n.s.
			eYFP 6-OHDA	30.91	6.20	15	5						eYFP saline vs M4 OE 6-OHDA	p=0.5285	n.s.
			M4 OE 6-OHDA	117.20	38.61	12	7								
		Slope (5-25 µA)	eYFP saline	25.11	2.30	12	6						eYFP saline vs eYFP 6-OHDA	p<0.0001	****
			eYFP 6-OHDA	11.13	1.40	15	5	p<0.0001	****		One-way ANOVA	Tukey	eYFP saline vs M4 OE 6-OHDA	p=0.2399	n.s.
			M4 OE 6-OHDA	20.78	1.71	12	7						eYFP 6-OHDA vs M4 OE 6-OHDA	p=0.0013	**

Figure	Section	Variables and units	Conditions	Mean	SEM	n	N	p-value	(*)	Data comparisons	Statistical test	Post-hoc test	Post-hoc comparisons	p-value	(*)
	D	Cylinder test Paw use (% contralateral)	eYFP saline	49.09	0.89		11	p<0.0001	****	Effect of treatment	Two-way ANOVA	Šídák	eYFP saline vs M4 OE saline	p=0.5129	n.s.
			M4 OE saline	46.39	0.97		18	p=0.5968	n.s.	Effect of group			eYFP 6-OHDA vs M4 OE 6-OHDA	p=0.9553	n.s.
			eYFP 6-OHDA	27.45	1.96		11	p=0.2341	n.s.	Interaction			eYFP saline vs M4 OE 6-OHDA	p<0.0001	****
			M4 OE 6-OHDA	28.50	2.06		14								
	E	Open field Mean velocity (cm/s)	eYFP saline	12.14	0.63		15	p<0.0001	****	Effect of treatment	Two-way ANOVA	Šídák	eYFP saline vs M4 OE saline	p=0.9468	n.s.
			M4 OE saline	11.72	0.66		17	p=0.9574	n.s.	Effect of group			eYFP 6-OHDA vs M4 OE 6-OHDA	p=0.9996	n.s.
			eYFP 6-OHDA	7.84	0.64		10	p=0.8001	n.s.	Interaction			eYFP saline vs M4 OE 6-OHDA	p<0.0001	****
			M4 OE 6-OHDA	7.76	0.53		15								
	F	Balance beam (19 mm) Number of slips	eYFP saline	0.11	0.07		9	p=0.0042	**	Effect of treatment	Two-way ANOVA	Šídák	eYFP saline vs M4 OE saline	p=0.9772	n.s.
			M4 OE saline	0.00	0.00		12	p=0.0129	*	Effect of group			eYFP 6-OHDA vs M4 OE 6-OHDA	p=0.0131	*
			eYFP 6-OHDA	1.40	0.81		5	p=0.0368	*	Interaction			eYFP saline vs M4 OE 6-OHDA	p=0.9812	n.s.
			M4 OE 6-OHDA	0.22	0.12		9								
	F	Balance beam (12 mm) Number of slips	eYFP saline	0.28	0.19		9	p=0.0618	n.s.	Effect of treatment	Two-way ANOVA				
			M4 OE saline	0.38	0.13		12	p=0.2553	n.s.	Effect of group					
			eYFP 6-OHDA	1.06	0.39		8	p=0.1201	n.s.	Interaction					
			M4 OE 6-OHDA	0.45	0.19		10								
		Balance beam (6 mm) Number of slips	eYFP saline	1.17	0.29		9	p<0.0001	****	Effect of treatment	Two-way ANOVA	Šídák	eYFP saline vs M4 OE saline	p=0.8396	n.s.
			M4 OE saline	1.54	0.36		12	p=0.3204	n.s.	Effect of group			eYFP 6-OHDA vs M4 OE 6-OHDA	p=0.1299	n.s.
			eYFP 6-OHDA	3.56	0.47		8	p=0.0497	*	Interaction			eYFP saline vs M4 OE 6-OHDA	p=0.0543	n.s.
			M4 OE 6-OHDA	2.45	0.32		10								
S4	A	M4-IPSC amplitude (pA)	Control saline	640.30	53.82	13	7	p=0.0369	*		Unpaired t-test				
		eStim intensity: 25μA	RGS4 cKO saline	770.00	32.99	24	11								
		Rise time (ms)	Control saline	61.38	2.46	9	5	p=0.0304	*		Mann-Whitney test				
		eStim intensity: 15μA	RGS4 cKO saline	66.75	0.99	28	11								
		Time constant (ms)	Control saline	94.96	4.40	10	6	p=0.0191	*		Unpaired t-test				
		eStim intensity: 15μA	RGS4 cKO saline	108.10	2.95	23	11								
	B	EC50 Oxo-M (μM)	Control saline	0.76	0.34	22	4-5	p=0.2552	n.s.		Unpaired t-test				
			RGS4 cKO saline	1.96	0.87	29	4-10								
	C	Firing frequency (Hz) ChI excitability						p<0.0001	****	Effect of injected current	Two-Way RM ANOVA with Geisser-Greenhouse's correction				
								p=0.6649	n.s.	Effect of group					
								p<0.0001	****	Effect of subject					
								p=0.0806	n.s.	Interaction					
	C	Firing frequency (Hz)	Control saline	2.81	0.55	22	3								
		Injected current: 50 pA	RGS4 cKO saline	2.82	0.64	21	4								
		Firing frequency (Hz)	Control saline	5.35	0.82	22	3								
		Injected current: 100 pA	RGS4 cKO saline	5.40	1.00	21	4								
		Firing frequency (Hz)	Control saline	7.05	0.98	22	3								
		Injected current: 150 pA	RGS4 cKO saline	7.44	1.21	21	4								
		Firing frequency (Hz)	Control saline	8.56	1.08	22	3								
		Injected current: 200 pA	RGS4 cKO saline	9.17	1.31	21	4								
		Firing frequency (Hz)	Control saline	9.28	1.15	22	3								
		Injected current: 250 pA	RGS4 cKO saline	10.33	1.31	21	4								
		Firing frequency (Hz)	Control saline	9.95	1.21	22	3								
		Injected current: 300 pA	RGS4 cKO saline	11.66	1.33	21	4								
	D	M4-IPSCs amplitude (pA)						p<0.0001	****	Effect of eStim intensity	Mixed-model ANOVA with Geisser-Greenhouse's correction	Holm-Šídák			
								p=0.0005	***	Effect of Group					
								p<0.0001	****	Interaction					
		30μA	Control saline	710.40	76.03	10	6						Control saline vs Control 6-OHDA	p=0.0017	**
			Control 6-OHDA	348.60	35.28	12	6						Control saline vs RGS4 cKO 6-OHDA	p=0.8199	n.s.
			RGS4 cKO 6-OHDA	687.70	62.55	19	8								
		25μA	Control saline	657.20	66.67	10	6						Control saline vs Control 6-OHDA	p=0.0012	**
			Control 6-OHDA	324.60	30.58	12	6						Control saline vs RGS4 cKO 6-OHDA	0.9625	n.s.
			RGS4 cKO 6-OHDA	652.91	61.27	19	8								
		20μA	Control saline	617.80	58.65	10	6						Control saline vs Control 6-OHDA	p=0.0004	***
			Control 6-OHDA	280.00	21.89	11	6						Control saline vs RGS4 cKO 6-OHDA	p=0.5535	n.s.
			RGS4 cKO 6-OHDA	570.00	53.63	19	8								
		15μA	Control saline	562.40	52.41	10	6						Control saline vs Control 6-OHDA	p=0.0002	***
			Control 6-OHDA	234.20	14.20	11	6						Control saline vs RGS4 cKO 6-OHDA	p=0.3659	n.s.
			RGS4 cKO 6-OHDA	494.70	51.31	17	8								
		10μA	Control saline	427.80	52.51	10	6						Control saline vs Control 6-OHDA	p=0.0014	**
			Control 6-OHDA	166.00	11.14	11	6						Control saline vs RGS4 cKO 6-OHDA	p=0.5014	n.s.
			RGS4 cKO 6-OHDA	380.10	45.82	17	8								
		5μA	Control saline	68.55	20.58	10	6						Control saline vs Control 6-OHDA	p=0.2243	n.s.
			Control 6-OHDA	30.95	9.00	11	6						Control saline vs RGS4 cKO 6-OHDA	p=0.8127	n.s.
			RGS4 cKO 6-OHDA	75.12	18.09	16	8								
		Slope (5-25 μA)	Control saline	27.35	3.07	10	6	p=0.0006	***		Kruskal-Wallis (ANOVA on ranks)	Dunn	Control saline vs control 6-OHDA	p=0.0019	**
			Control 6-OHDA	13.12	1.23	11	6						Control saline vs RGS4 cKO 6-OHDA	p>0.05	n.s.
			RGS4 cKO 6-OHDA	25.99	2.76	17	8						Control 6-OHDA vs RGS4 cKO 6-OHDA	p=0.0026	**
	E	Cylinder test Paw use (% contralateral)	Control saline	50.28	0.83		19	p<0.0001	****	Effect of treatment	Two-way ANOVA	Šídák	Control saline vs RGS4 cKO saline	p=0.9959	n.s.
			RGS4 cKO saline	54.92	2.70		22	p=0.8403	n.s.	Effect of group			Control 6-OHDA vs RGS4 cKO 6-OHDA	p=0.9959	n.s.
			Control 6-OHDA	15.03	2.99		24	p=0.141	n.s.	Interaction			Control saline vs RGS4 cKO 6-OHDA	p<0.0001	****
			RGS4 cKO 6-OHDA	11.84	2.94		21								
	F	Open field Mean velocity (cm/s)	Control saline	10.27	0.51		21	p<0.0001	****	Effect of treatment	Two-way ANOVA	Šídák	Control saline vs RGS4 cKO saline	p=0.0027	**
			RGS4 cKO saline	7.65	0.69		21	p=0.2292	n.s.	Effect of group			Control 6-OHDA vs RGS4 cKO 6-OHDA	p=0.2408	n.s.
			Control 6-OHDA	4.06	0.36		22	p=0.0005	***	Interaction			Control saline vs RGS4 cKO 6-OHDA	p<0.0001	****
			RGS4 cKO 6-OHDA	5.37	0.55		20								
	G	Balance beam (19 mm) Number of slips	Control saline	0.00	0.00		15	p=0.021	*	Effect of treatment	Two-way ANOVA	Šídák	Control saline vs RGS4 cKO saline	p>0.05	n.s.
			RGS4 cKO saline	0.00	0.00		15	p=0.021	*	Effect of group			Control 6-OHDA vs RGS4 cKO 6-OHDA	p=0.0056	**
			Control 6-OHDA	0.95	0.53		11	p=0.021	*	Interaction			Control saline vs RGS4 cKO 6-OHDA	p>0.05	n.s.
			RGS4 cKO 6-OHDA	0.00	0.00		17								
	G	Balance beam (12 mm) Number of slips	Control saline	0.03	0.03		15	p=0.0008	***	Effect of treatment	Two-way ANOVA	Šídák	Control saline vs RGS4 cKO saline	p=0.9675	n.s.
			RGS4 cKO saline	0.30	0.17		15	p=0.0015	**	Effect of group			Control 6-OHDA vs RGS4 cKO 6-OHDA	p=0.0001	***
			Control 6-OHDA	3.27	1.19		11	p=0.0043	**	Interaction			Control saline vs RGS4 cKO 6-OHDA	p=0.9899	n.s.
			RGS4 cKO 6-OHDA	0.21	0.15		17								
		Balance beam (6 mm) Number of slips	Control saline	1.60	0.32		15	p=0.1459	n.s.	Effect of treatment	Two-way ANOVA				
			RGS4 cKO saline	1.40	0.22		15	p=0.6881	n.s.	Effect of group					
			Control 6-OHDA	2.29	0.91		7	p=0.9835	n.s.	Interaction					
			RGS4 cKO 6-OHDA	2.11	0.46		19								
S5	A	Cylinder test	Control 6-OHDA	17.39	2.19		21	p=0.1528	n.s.		Mann-Whitney				
		Paw use (% contralateral)	RGS4 cKO 6-OHDA	20.84	3.02		20								
	B	M4-IPSC amplitudes (pA)						p<0.0001	****	Effect of eStim intensity	Mixed-model ANOVA with Geisser-Greenhouse's correction	Holm-Šídák			
								p<0.0001	****	Effect of Group					
								p<0.0001	****	Interaction					
		30μA	Control 6-OHDA+L-DOPA	256.60	30.38	18	5							p<0.0001	****
			RGS4 cKO 6-OHDA+L-DOPA	619.30	32.88	18	4								
		25μA	Control 6-OHDA+L-DOPA	233.20	29.40	18	5							p<0.0001	****
			RGS4 cKO 6-OHDA+L-DOPA	593.70	32.82	18	4								
		20μA	Control 6-OHDA+L-DOPA	188.40	29.64	18	5							p<0.0001	****
			RGS4 cKO 6-OHDA+L-DOPA	517.50	30.26	17	4								
		15μA	Control 6-OHDA+L-DOPA	144.40	28.09	18	5							p<0.0001	****
			RGS4 cKO 6-OHDA+L-DOPA	458.60	31.27	17	4								
		10μA	Control 6-OHDA+L-DOPA	95.26	22.30	18	5							p<0.0001	****
			RGS4 cKO 6-OHDA+L-DOPA	346.20	37.55	17	4								
		5μA	Control 6-OHDA+L-DOPA	20.95	12.34	18	5							p=0.0233	*
			RGS4 cKO 6-OHDA+L-DOPA	74.15	18.44	17	4								
		Slope (5-25 μA)	Control 6-OHDA+L-DOPA	10.35	1.32	18	5	p<0.0001	****		Unpaired t-test				
			RGS4 cKO 6-OHDA+L-DOPA	24.03	1.83	17	4								
	C	Mean velocity (cm/s)	Control LID	3.95	0.16		15	p=0.0575	n.s.		Unpaired t-test				
			RGS4 cKO LID	3.50	0.16		14								
